# Chimeric NANOG repressors inhibit glioblastoma growth in vivo in a context-dependent manner

**DOI:** 10.1038/s41598-019-39473-y

**Published:** 2019-03-07

**Authors:** Monika Kuciak, Christophe Mas, Isabel Borges, Pilar Sánchez-Gómez, Ariel Ruiz i Altaba

**Affiliations:** 1 Department of Genetic Medicine and Development, University of Geneva Medical School, Rue Michel Servet 1, CH-1211, Geneva, Switzerland; 2 Neuro-oncology Unit, Instituto de Salud C.III-UFIEC, Madrid, Spain; 3 Present Address: Oncotheis Sàrl. 18 chemin des Aulx, CH-1228 Plan-Les-Ouates, Geneva, Switzerland

## Abstract

Targeting stemness promises new therapeutic strategies against highly invasive tumors. While a number of approaches are being tested, inhibiting the core transcription regulatory network of cancer stem cells is an attractive yet challenging possibility. Here we have aimed to provide the proof of principle for a strategy, previously used in developmental studies, to directly repress the targets of a salient stemness and pluripotency factor: NANOG. In doing so we expected to inhibit the expression of so far unknown mediators of pro-tumorigenic NANOG function. We chose NANOG since previous work showed the essential requirement for NANOG activity for human glioblastoma (GBM) growth in orthotopic xenografts, and it is apparently absent from many adult human tissues thus likely minimizing unwanted effects on normal cells. NANOG repressor chimeras, which we name NANEPs, bear the DNA-binding specificity of NANOG through its homeodomain (HD), and this is linked to transposable human repressor domains. We show that *in vitro* and *in vivo*, NANEP5, our most active NANEP with a HES1 repressor domain, mimics knock-down (kd) of NANOG function in GBM cells. Competition orthotopic xenografts also reveal the effectiveness of NANEP5 in a brain tumor context, as well as the specificity of NANEP activity through the abrogation of its function via the introduction of specific mutations in the HD. The transcriptomes of cells expressing NANEP5 reveal multiple potential mediators of pro-tumorigenic NANEP/NANOG action including intercellular signaling components. The present results encourage further studies on the regulation of context-dependent NANEP abundance and function, and the development of NANEP-based anti-cancer therapies.

## Introduction

Glioblastoma (GBM) currently has an average survival period of ~15 months from diagnosis^1^ due to the impossibility to cure the patient by removing all affected tissue. Even after surgery, chemo- and radiotherapy residual cancer stem cells can regenerate the tumor and spread into the healthy brain^1–5^. Targeting these cells is challenging given their high resistance to conventional treatments. However, it may be possible to halt their self-renewal and thus persistence by blocking essential cancer stemness determinants.

NANOG is a homeodomain protein involved in the maintenance of embryonic stemness and self-renewal^6–8^. It is highly expressed in the mouse and human blastocyst’s inner cell mass as well as in embryonic stem cells (ESCs), in which it has been shown to be involved, yet dispensable, for maintenance^9^. With a few exceptions, NANOG is not expressed in adult human tissues (https://www.proteinatlas.org/ENSG00000111704-NANOG/tissue; http://gliovis.bioinfo.cnio.es/ NANOG in TCGA-GBM dataset)^6,10^, although it has been detected in basal cells of stratified epithelia in mice^11^. It is thus notable that it is expressed in multiple human cancers of diverse origins, including those of the brain, colon, prostate and stomach^12–17^. In gliomas, we have previously shown that NANOG is essential for growth and invasiveness^18^. NANOG is thus a superb target for anticancer therapies, which should have few if any on-target side effects. However, how to inhibit its function remains a challenge.

**Figure 1:**
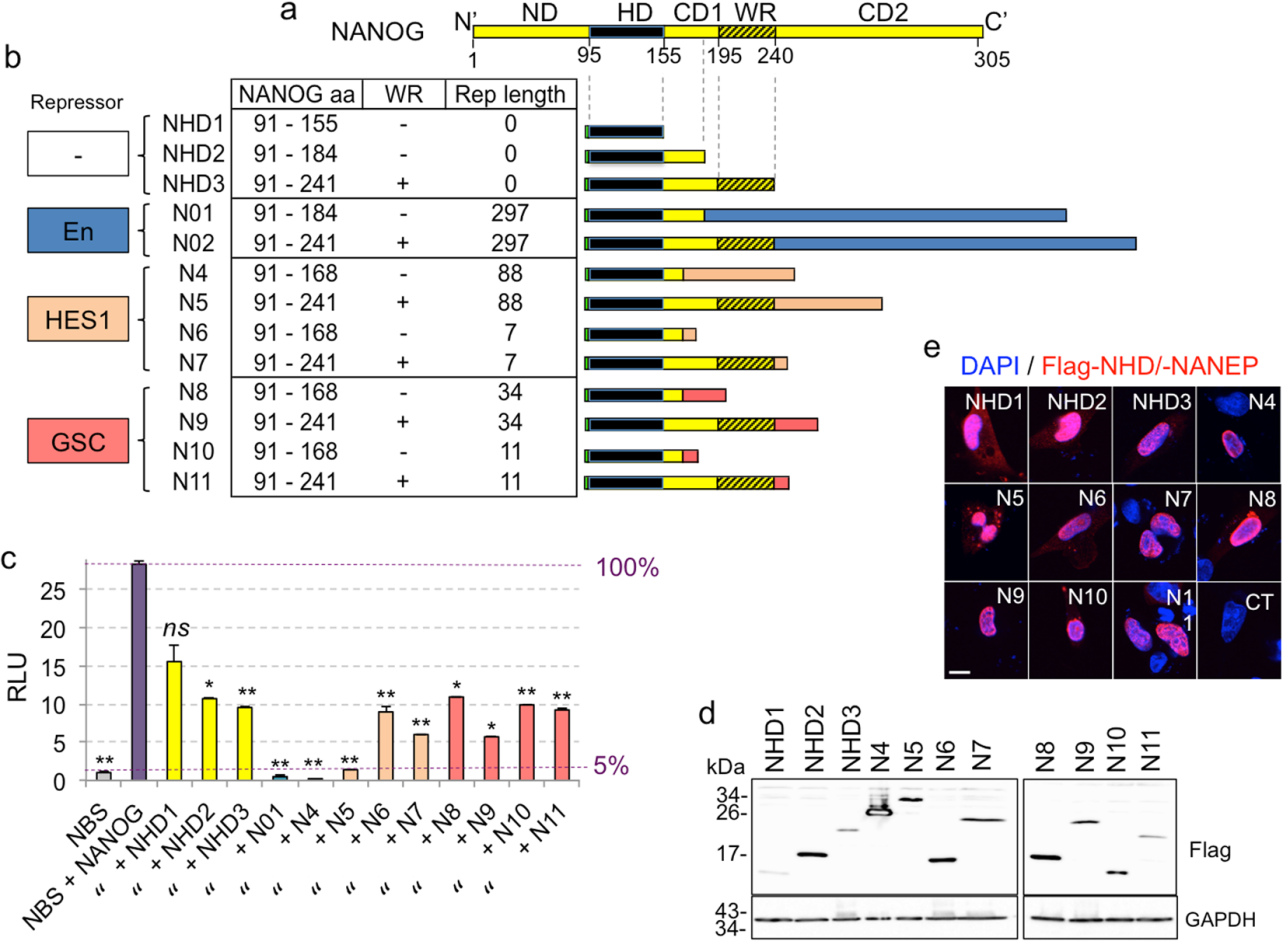
Structure and activity of NANEPs. (**a**) Linear box diagram of NANOG domains from the N-terminus (N’, left) to the C-terminus (C’, right): ND- N-terminal domain, HD – Homeodomain, CD1- linker, WR- dimerization domain, CD2- C-terminal transactivation domain. Amino acids’ positions counted from the N-terminus are shown below the scheme. (**b**) Table and schemes describing constructs used in the study. The type of repressor domain used is annotated on the left in colored boxes: Drosophila Engrailed (En) in blue, human HES1 in orange and human GSC (GOOSCOID) in pink. The table shows the segments of NANOG present in the various NANEPS (N01-N11), presence (+) or absence (−) of the dimerization WR domain, and the lengths of each repressor domain used. Also included are the NANOG homeodomain only (NHD1-3) constructs. (**c**) Quantification of NANOG binding site (NBS) reporter construct activity in Firefly/Renilla luciferase relative light units (RLU) in 293T cells from the testing of the different constructs shown in panel (**b**). The activity in NBS (first bar) represents background noise (5% of maximum obtained with NANOG, equated to 100%). The activtiy in NBS + NANOG served as control and was used to calculate *P values* for all conditions. Constructs annotated as in panel (b) where N = NANEP. Error bars are SEMs. **p < 0.05; ***p < 0.01; ns = not significative (p > 0.05). (**d**) Western blot showing the expression of flag-tagged control (NHD1-3) and NANEP (N4-N11) constructs. GAPDH was used as a loading control. Size of protein marker bands (in kDa) are shown on the left of each blot. The expected molecular sizes for the constructs are: NHD1: 10 kDa; NHD2: 13 kDa; NHD3: 19 kDa; N4: 21 kDa; N5: 28 kDa; N6: 12 kDa; N7: 20 kDa; N8: 15 kDa; N9: 23 kDa; N10: 13 kDa; N11: 20 kDa. (**e**) Expression and cellular localization of NHDs and NANEPs. The confocal microscopy single slice (4 µm) images show merged signals of flag-tagged proteins (red) and DAPI-stained nuclei (blue) in U87 cells 36 h after transfection. Similar results were obtained in U251 cells (not shown). Control (CT) here is NHD1- transfected cells labeled only with the RITC-coupled secondary antibody. Scale bar = 15 μm.

Structurally, NANOG is a 305 aa-long transcription factor with a 60 aa homeodomain (HD) (Fig. 1a). C-terminally to the HD there is the CD1 linker region followed by a 45 aa tryptophan rich (WR) dimerization domain, and a larger CD2 transactivation region (Fig. 1a)^19,20^.

*NANOG (NANOG1)* has 11 pseudogenes and at least *NANOGP4*, *P5* and *P8* are expressed in cancer cells^17,18,21–23^. Importantly, *NANOG1* and *NANOGP8*, which is a retrogene, encode NANOG proteins that differ by 1-2 conservative amino acid changes^10,14,24^ indicating that functional NANOG protein can be made from two genes in human cancer cells^14,18,21^.

How NANOG functions is not clear. In different systems it can work as a transcriptional activator or repressor and the endogenous protein has been shown to bind many DNA sites in ESCs^25^. Moreover, NANOG function and its regulated genes seem to be highly context-dependent^26^. For example, NANOG promotes but is not required for colon cancer xenograft growth^14,27^. In contrast, it is essential for GBM growth *in vivo*^18^ and for directly seeded colon cancer metastases in mice^27^. Similarly, its overexpression leads to different outcomes depending on the tumor type: It is insufficient on its own to enhance colon cancer xenograft growth^27^ or to provoke breast tumor initiation in transgenic mice^28,29^, although it results in hyperplasia in the oesophagus^11^ and the formation of squamous cell carcinomas in skin^30^. The bases for this context-dependency are unknown.

Blockade of NANOG function has been achieved in preclinical models through RNA interference targeting both *NANOG* and *NANOGP8* with siRNAs and shRNAs^14,18,31^. However, few such approaches have already reached clinical trials^32^. There is also with dearth of anti-NANOG inhibitory small molecules although aspirin has been suggested to affect NANOG protein stability *in vitro*^33^. Here we have taken an alternative and parallel approach to target NANOG function through the use of chimeric repressors.

In this study we aimed to provide the proof of principle that a constitutive dominant-negative NANOG chimeric protein - NANEP for NANOG repressor protein - is efficient to inhibit GBM growth. In doing so, we assumed its known positive transcriptional activity^8,18^ would be essential in cancer. We therefore set three benchmarks an active and potentially useful NANEP should fulfill: i- It should mimic the reported phenotype of *shNANOG* in GBM cells, inhibiting tumor growth *in vivo* and clonogenic growth *in vitro*^18^; ii- specific mutations reported to abolish DNA binding of mouse Nanog^34^ should abolish anti-tumor effects *in vivo*; iii- NANEP-regulated genes should include at least some genes reported to bind NANOG in ESCs^25^.

## Results

As an initial step towards the construction of NANEPs we used the 60 aa NANOG HD (NHD) - which confers DNA binding-specificity - plus the CD1 region present in a full length transcript, but not in an alternative splice variant^20^ (Fig. 1a). In addition, we used this 94 aa portion plus the adjacent WR dimerization domain (Fig. 1a) to enhance function if NANOG were to act as a dimer in cancer cells as it does in ESCs^35,36^. These two segments of NANOG (HD-CD1 and HD-CD1-WR) were first fused to the extended 297 aa Drosophila Engrailed protein repressor domain (EnR) yielding NANEP01 and NANEP02 (Fig. 1b). We chose EnR, which interacts with the co-repressor factor Groucho (Gro)^37,38^, as it has been widely used in developmental studies to confer repressor function to chimeric proteins^37,39–44^.

To measure NANEP activity we built a NANOG binding site->Firefly luciferase construct (NBS-Luc) carrying three tandem copies of the canonical NANOG binding site CGCCGATTAAG (Fig. S1a)^6^. Importantly, NANEP01 and NANEP02 (N01 and N02 in Fig. 1b), equally repressed the NBS-Luc reporter activated by co-transfected NANOG (Fig. S1b; Fig. 1c). This first result prompted us to design additional NANEPs with smaller human repressor domains previously proven to function in heterologous systems.

We thus built N-terminally FLAG-tagged long and short NANEPs using two different repressor domains at the C-terminus (Fig. 1b). These were from the human HES1 and GOOSECOID (GSC) proteins, utilizing reported short repressor domains^38,45,46^ as well as longer versions in case adjacent additional sequences might be required for structural stability and/or folding (Figs 1b and S2). The repressor domains of both HES1 and GSC contain binding sites for Gro/TLE co-repressors^38,47^. The smallest repressor domain version consisted of only 8 aa containing WRPW motif of HES1, known to be able to transfer repressor function to chimeric proteins^46^ (N6 and N7 in Fig. 1b).

These various human HES1 and GSC repressor domains were fused to the NHD in two flavors (Fig. 1b): i- the 77 aa-long NHD-CD1 segment; and ii- the 150 aa-long NHD-CD1-WR segment containing the dimerization domain reported to be involved in NANOG dimerization and stability enhancement^35,36^.

As controls for the possible negative action of NANEPs due only to physical interference of full-length NANOG rather than active repression, we built three versions of the NHD without any repressor domains: one containing only the NHD (60aa) named NHD1, the NHD-CD1 segment named NHD2, and NHD-CD1-WR segment named NHD3 (Figs 1b and S2).

Dual luciferase assays in human 293T cells with the NBS->Luc and renilla controls with each co-transfected NANEP revealed varying activities. NBS->Luc alone yielded background levels of 5% of the maximal activation achieved by co-transfected NANOG (Fig. 1c). The small homeodomain-only NHD1 construct showed no statistically significative reduction in reporter activity driven by NANOG, although the larger NHD2 and NHD3 reduced activity to 58% and 65%, respectively, indicating the level of passive repression.

Ten NANEPs were then tested individually and simultaneously on NBS->Luc with co-expressed NANOG. Whereas NANEPs 01 and 02 abolished reporter activation by co-expressed NANOG as expected (see above), NANEPs 6,8,10 and 11 showed similar activity as the NHD2 and NHD3 controls (Figs 1b,c and S1b), indicating their lack of active repressive function. NANEP7 and NANEP9, however, reduced reported activity by ~80% and the best results were obtained with NANEP4 and NANEP5, which fully repressed NANOG-driven activity to background levels (i.e. by 95%), mimicking NANEP01 and 02 (Figs 1b,c and S1b).

Transfection of human 293T cells with equal amounts of plasmid vectors harboring EF1α regulatory elements revealed the presence of the expected sizes for NANEP proteins by Western blotting. It is noticeable that the levels of abundance of mature protein varied, although this, on its own, does not seem able to account for all functional differences, as NANEP7, for instance, is a weaker repressor than NANEP5 but both showed similar normalized protein levels (Figs 1c,d and S1c). Differences in activity were also not related to major changes in subcellular localization as all NANEPs tested and the three NDH controls showed strong nuclear signals (Fig. 1e).

**Figure 2:**
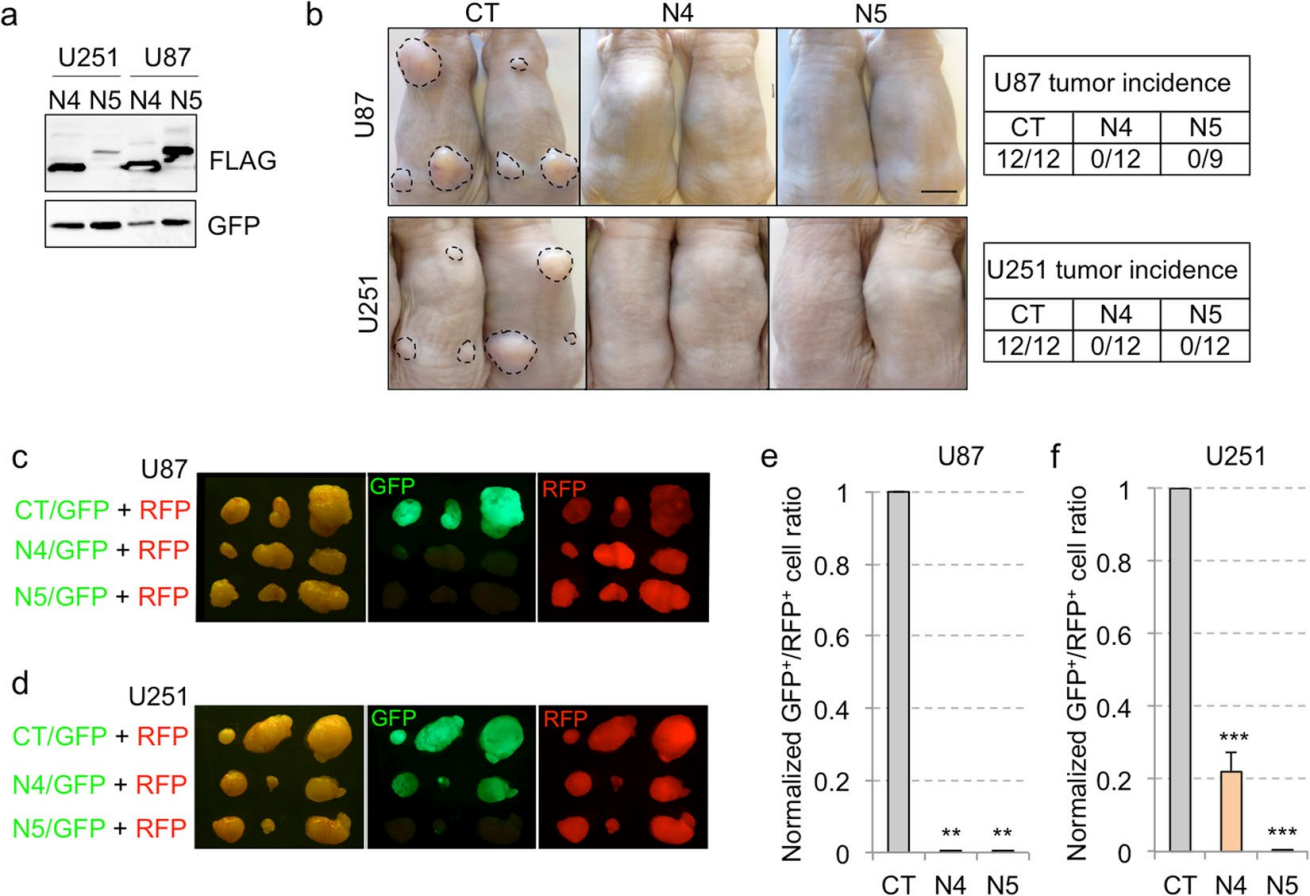
NANEP4 and NANEP5 are efficient inhibitors of subcutaneous GBM growth. (**a**) Western blot of the expression of NANEP4 (N4) and NANEP5 (N5) in U251- and U87-transduced cells. Here GFP was used as a lentiviral infection and loading control. (**b**) Images showing the backs of NUDE mice focally injected in three sites with U87 (upper panels) or U251 (lower panels) cells infected with control (CT), NANEP4 (N4) or NANEP5 (N5) expressing lentivectors. Dashed lines encircle the tumors. Note the absence of tumor growth from NANEP-expressing cells. Tumor incidence for each cell type is shown in tables on the right side. n = 12 tumors for each condition, except for U87 N5 where n = 9. (**c**,**d**) Representative images of dorsal views of tumors seen under visible light (left panels), and with GFP (middle panels) or RFP fluorescence (right panels). U87 (**c**) and U251 (**d**) cells were infected with vectors expressing GFP and RFP as indicated on the left side. CT: vector alone control, N4: NANEP4, N5: NANEP5. (**e**,**f**) Quantification of the GFP^+^/RFP^+^ cell ratios in U87 (**e**) and U251 (**f**) tumors after whole tumor dissociation and FACS analyses. Ratios form control tumors (CT) were normalized to 1. Error bars are SEM. n = 12, **p < 0.05; ***p < 0.01. Scale bar = 1 cm for (**b**–**d**).

These results allowed us to focus on NANEP4 and NANEP5 to continue the exploration of the principle that NANEPs can be efficient anti-cancer agents. Appropriate lentivectors for stable expression were then made and used to transduce human U87 and U251 GBM cells with NANEP4 or NANEP5, yielding stable NANEP proteins (Fig. 2a). As expected, they were shown to inhibit the activation of luciferase from a NHD->Luc construct by co-expressed NANOG in U251 glioblastoma (GBM) cells (Fig. S3).

As a first *in vivo* test for the anti-cancer function of NANEPs we independently injected U87 and U251 cells expressing NANEP4 or NANEP5 into the flanks of immunocompromised NUDE mice. Control cells carrying empty lentivectors yielded tumors that could be visualized and measured (Fig. 2b). In contrast, neither cell type with neither NANEP4 nor NANEP5 formed any tumors (Fig. 2b), taking the animals at the same time as the controls (45 days after cell injection). Mice xenografted with NANEP4- or NANEP5-expressing cells showed no signs of disease.

As a second *in vivo* test we used a red/green competition assay we developed to monitor cell viability in a tumorigenic context^18,48^. Here, U87 or U251 cells were transduced with either GFP^+^ or RFP^+^ lentivectors. The GFP^+^ (green) cells also received the NANEP lentivectors, whereas the RFP^+^ (red) cells received only the control vectors. Red cells thus work as controls ensuring tumor growth in which the green cells can develop and proliferate, or not, but always in the presence of viable cancer cells that build a tumor microenvironment. As expected, all tumors grew (with small statistically insignificant differences, p > 0.2) and all had red cells (Fig. 2c,d). NANEP4 or NANEP5 were equally effective in eliminating any green cell growth inside the U87 tumors (Fig. 2c). However, NANEP4 was only partially effective in U251 grafts and green cells could be visualized in the tumor mass (Fig. 2d). Quantification of the GFP^+^/RFP^+^ ratios by FACS of tumor dissociated cells clearly showed the partial activity of NANEP4 in U251 cells (Fig. 2e,f). Given these results and the apparent context-dependency of NANEP4, we focused hereafter on NANEP5, which harbors a fragment of NANOG from the HD to the WR domain (NHD-CD1-WR; Figs 1a and S2).

**Figure 3:**
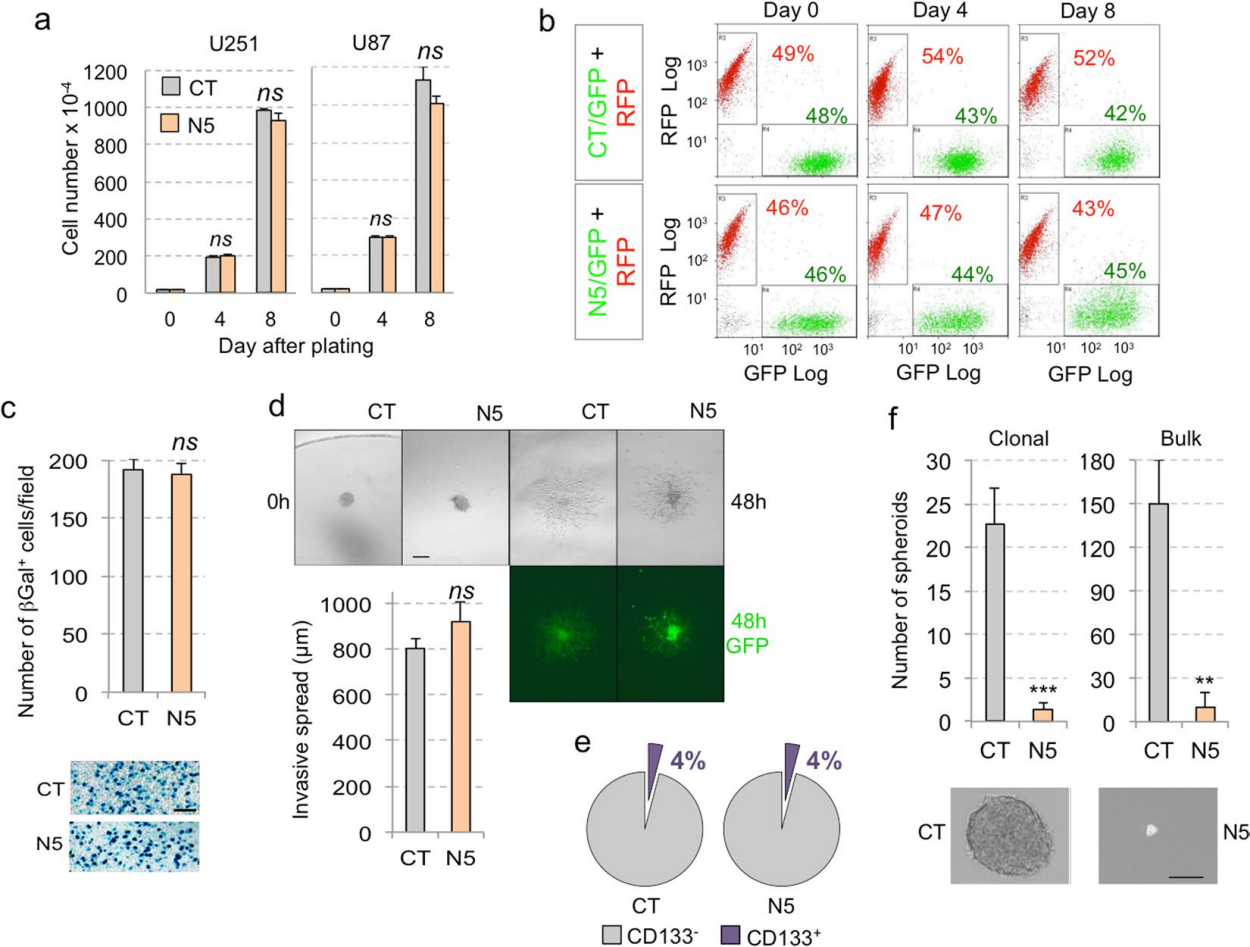
NANEP5 activity *in vitro*. (**a**) Quantification of U251 (left) and U87 (right) GBM cell numbers at different time points during vitro culture. CT: control; N5: NANEP5; Error bars are SEM. ns: not significative (p > 0.05). (**b**) FACS plots illustrating analyses at different time points of co-culture of U251 cells infected either with: GFP or N5/GFP, and RFP vectors. GFP^+^ and RFP^+^ cell percentage is annotated on each plot in green or red color, respectively. (**c**) Left) Quantification of U251 βGal^+^ cells in transfilter assays having migrated into the filter. Right) Representative pictures of the membranes after fixation and X-Gal staining for both control (CT) and NANEP5 (N5) cell populations. Error bars are SEM. ns: not significative (p > 0.05). n = 6 for both conditions. Scale bar = 200 μm. (**d**) Left) Quantification of the invasive spread into a collagen cushion of U251 cells. Error bars are SEM. ns: not significative (p > 0.05). Right) Images of colonies pictured just after plating (0 h) and 48 h later under visible light (left and middle panels) and GFP fluorescence (right panels). n = 17 for CT; n = 7 for N5. Scale bar = 250 μm. (**e**) Pie charts illustrating the percentage of MACS sorted CD133^+^ populations in U251 cells infected either with control (CT) or NANEP5 (N5)-expressing vectors. n = 3 for each. (**f**) Left) Histograms showing the quantification of U251 spheroids formed under clonal conditions (left) and in the bulk (right). **p < 0.05; ***p < 0.01. Pictures on the right show a representative image of a control (CT) spheroid and a rare small remnant from NANEP5-expressing (N5) cells. Scale bar = 120 μm.

Given that *NANOG* kd has no effect on GBM cell proliferation in 2D culture^18^ we tested for any possible effect of NANEP5 *in vitro*. Cell counts over several passages revealed that NANEP5 expression did not hamper U87 or U251 tumor cell proliferation (Fig. 3a). Similarly, *in vitro* red/green competition assays with NANEP5 in U251 cells failed to reveal any effect, maintaining the red/green ratio over two consecutive passages (Fig. 3b). NANEP5 was also ineffectual on U251 transwell migration (Fig. 3c) and on collagen invasion *in vitro* (Fig. 3d). Finally, whereas the number of putative U251 CD133^+^ colon cancer stem cells (4%) was unaffected (Fig. 3e), the number of clonal spheroids was drastically reduced upon NANEP5 expression either upon diluted single cell or in bulk conditions (Fig. 3f), much as reported for NANOG kd^18,27^. These results suggest that NANEP5 does not have an effect on cell proliferation in 2D culture or on the number of putative stem cells, but rather that it inhibits the self-renewal capacity of these cells under spheroid-forming conditions. *In vitro*, NANEP5 thus mimics *NANOG* kd.

**Figure 4:**
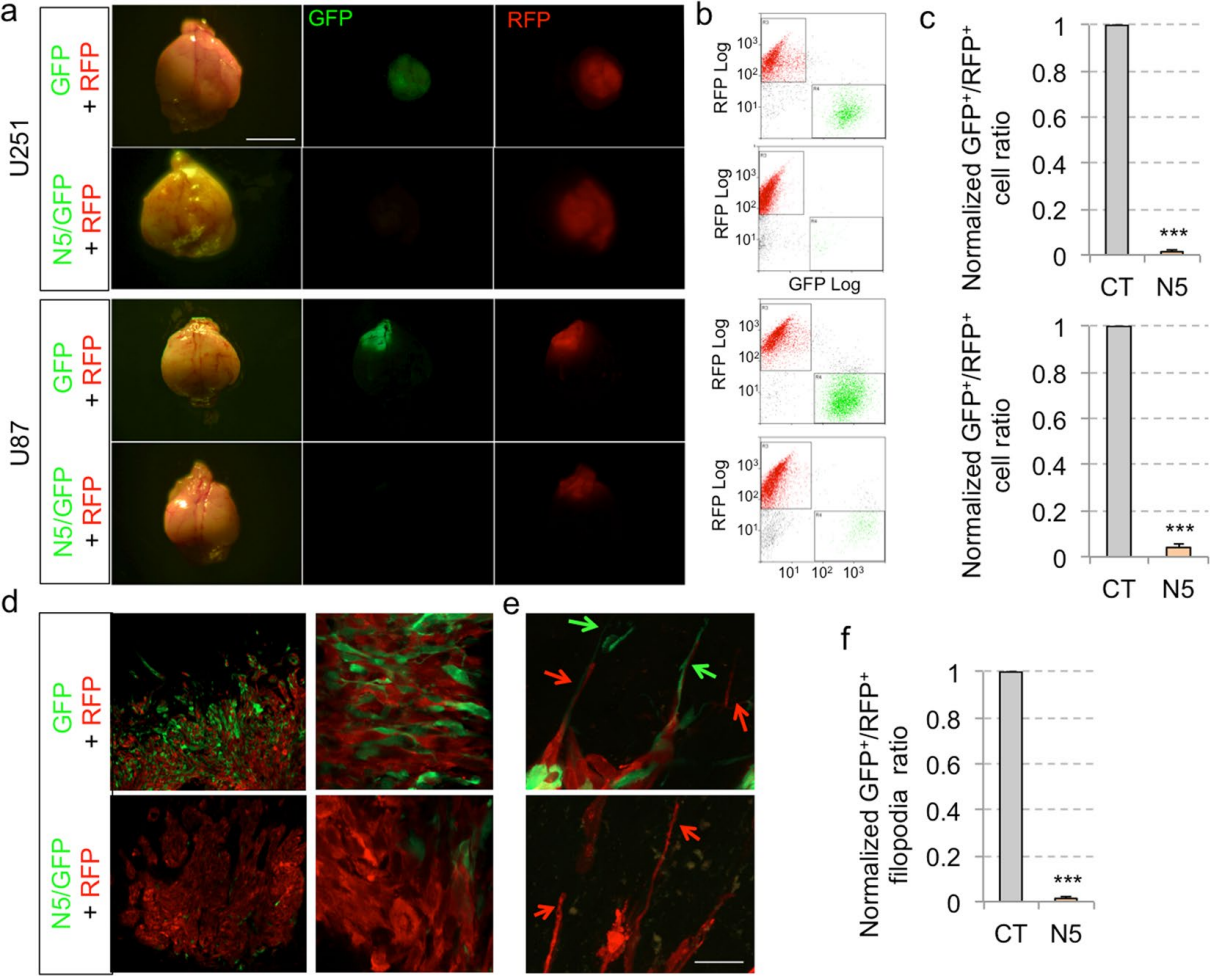
NANEP5 obliterates the growth of orthotopic GBM xenografts in a viable tumor context. (**a**) Representative images of dorsal views of dissected mouse brains after orthotopic brain xenotransplantation of U251 (top panels) and U87 (bottom panels) infected with GFP and RFP expressing lentivectors as indicated. The GFP lentivector was used alone as control (GFP) or with NANEP5 (N5/GFP). Pictures were taken under visible (left panels) and fluorescent light (GFP and RFP, middle and right panels, respectively). Scale bar = 4 mm. (**b**) FACS plots showing GFP^+^/RFP^+^ cells ratios after dissociation of the entire brain tumors shown in the same row in A. Note the absence of green GFP^+^ cells in N5/GFP U251 grafts but the presence of few GFP^+^ cells in N5/GFP U87 grafts. (**c**) Quantification of the GFP^+^/RFP^+^ cells ratios in U251 (upper histogram) and U87 (lower histogram) orthotopic GBM tumors after whole tumor dissociation and FACS analysis. Ratios form control tumors were normalized to 1. Error bars are SEM. ***p < 0.01. U251 experiment: n = 16 for control (CT), n = 14 for NANEP5 (N5) condition; U87 experiment: n = 4 for both conditions. (**d**) Confocal images of brain slices from mice taken 3 weeks after orthotopic grafting of U251 red and green cells. Panels show representative low (left) and high (right) magnification images. Note the abundance of viable GFP^+^ cells in control (CT, upper panel) and their absence in NANEP5/GFP (N5/GFP, lower panel) tumors. A few weakly, mostly rounded, GFP^+^ cells could be observed in the tumor mass (lower right panel). Scale bar = 400 μm for left panels, 50 μm right panels. (**e**) Confocal images of control and NANEP5 expressing U251 grafts as in (**d**) showing edges of the tumor mass. In control (GFP + RFP) tumors visible cell filopodia (arrows in upper panel) can be observed invading adjacent healthy brain tissue (black area). No equivalent GFP^+^ filopodia were detected in NANEP5-expressing U251 cells. In contrast, RFP+ filopodia were abundant (arrows in lower panel). Scale bar = 65 μm for both panels. (**f**) Quantification of the ratio of GFP^+^ filopodia/RFP^+^ filopodia at the edge of the tumor mass (as shown in **e**) observed in a 350 μm–long edge segment. This length was chosen for quantification purposes. The total number of filopodia per 350 µm segment in control tumors was 28. n = 10 counted 350 µm segments for each condition.

As a final test for the *in vivo* efficacy of NANEP5 we performed *in vivo* orthotopic xenografts using red/green competition assays with U87 cells, which are rarely invasive, as well as with U251 cells, which are highly invasive^49^. Whereas red control U87 or U251 cells grew and formed intracerebral tumors, green cells expressing NANEP5 were absent or drastically reduced in numbers (Fig. 4a–c). Control U251 grafts showed both green and red cells at the tumor center and at the periphery (Fig. 4d). Red and green control cells with long filopodia (>3 nuclear diameters) were present at the invasive fronts indicative of tumor cell penetration into the surrounding brain parenchyma (Fig. 4e). In contrast, NANEP5^+^/GFP^+^ cells were completely absent from the invading front, which was solely composed of red control cells (Fig. 4e,f).

To address the efficacy of NANEP5 in an established tumor we built a conditional doxycycline (DOX)-inducible lentivector system with rtTA derived from a second lentivector^27,50^. Induction of such NANEP5^conditional^ system with DOX *in vitro* resulted in the strong upregulation of the expression of *NANEP5* mRNA (Fig. S4a). Orthotopic grafting of green U251 cells harboring the NANEP5^conditional^, GFP system along with sibling red U251 cells (transduced with a RFP-expressing lentivector) was first performed without DOX as control (Fig. S4b). Surprisingly, analyses of intracranial grafts under these conditions resulted in the lack of green (GFP-only) cells (Fig. S4b). This indicated that the weak leakiness of the system: 100-fold lower in −DOX vs. +DOX conditions and on average 56-fold lower than with the straight system (Fig. S4a) allows enough NANEP5 to be made to abolish tumor growth. Thus, even though we could not conditionally turn on NANEP5 activity in growing tumors, the data suggests the NANEP5 is highly active.

**Figure 5:**
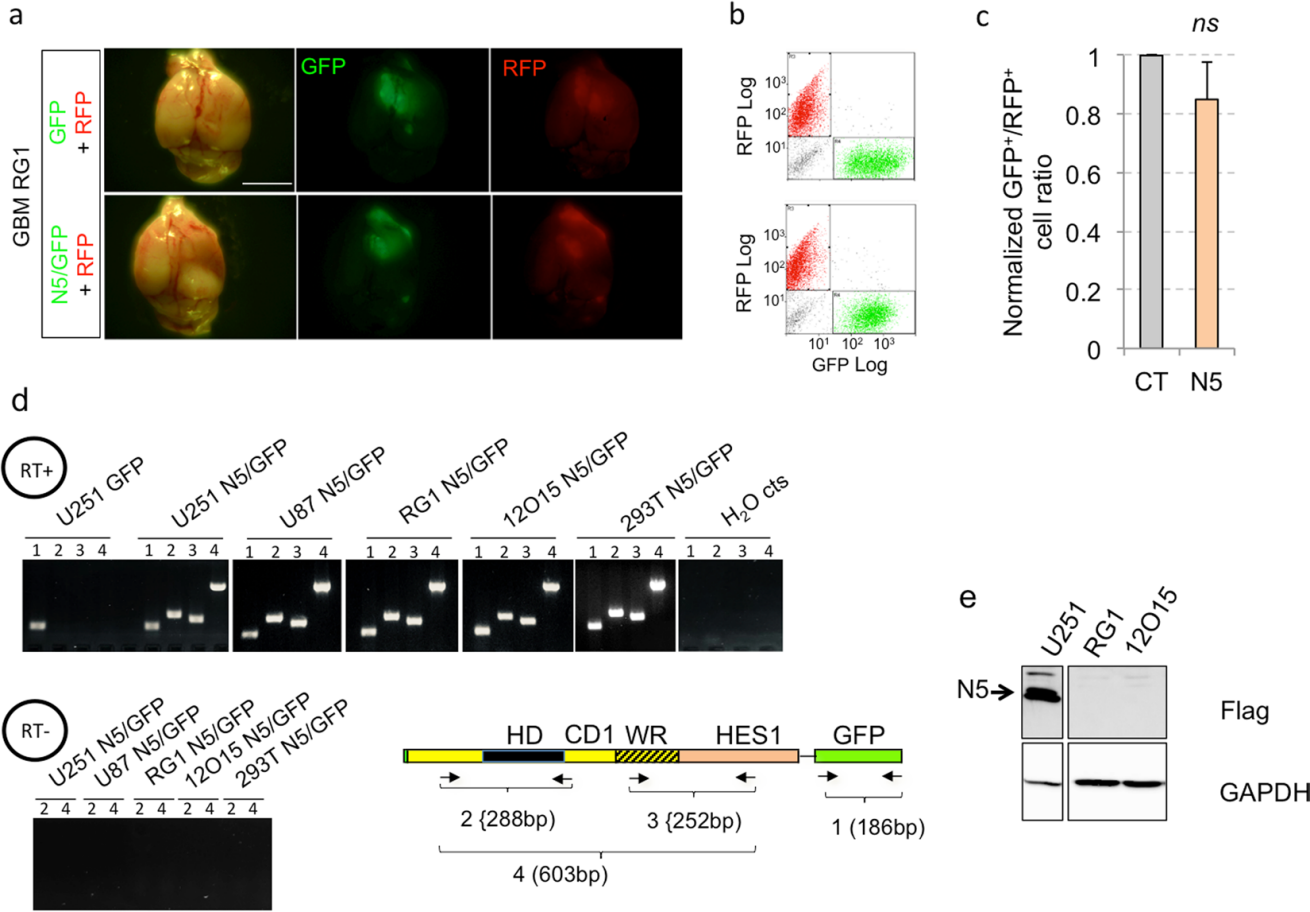
Absence of stable NANEP5 protein in two primary GBMs. (**a**) Representative images of dorsal views of dissected mouse brains after orthotopic xenotransplantation of primary RG1 GBM red and green cells (infected with GFP^+^ or RFP^+^ lentivectors as indicated on the left). Pictures show dorsal views and were taken under visible (left panels) and fluorescent light (GFP and RFP, middle and right panels, respectively). Scale bar = 4 mm for all panels. (**b**) FACS plots showing GFP^+^/RFP^+^ cells ratios after dissociation of whole RG1 brain tumors shown in the same rows in panel (**a**). (**c**) Quantification of GFP^+^/RFP^+^ cells ratios in RG1 tumors after whole tumor dissociation and FACS analysis. Ratios form control tumors were normalized to 1. Error bars are SEM. ns = not significative (p > 0.05). n = 4 grafts per condition. (**d**) Mapping transgene transcription. RT-PCR results shown were performed on RNA extracted from 5 different cell types (annotated above the pictures of agarose gels) infected with *GFP*-expressing empty lentivector as control (GFP) or *GFP* and *NANEP5*-expressing lentivectors (N5/GFP) using four different pairs of primers as noted in the box diagram. Negative controls (cts) include: control *GFP*^+^ infected U251 cells, H_2_O controls and reverse transcriptase negative (RT-) reactions (lower panel). Primers alignments within *NANEP5* and *GFP* regions as well as the expected sizes of PCR products (1–4) are shown on the box diagram. Note that all *NANEP5* regions are expressed in all *NANEP5*-transduced cell types. (**e**) Western blots showing the lack of NANEP5 (N5) protein expression in two primary GBMs (RG1 and 12O15), which contrasts to its clear expression in U251 cells (arrow). GAPDH was used as a loading control.

We also sought to test the activity of NANEP5 in primary GBM tumor samples from patients using these *in vivo* red/green orthotopic competition assays. Two independent primary GBM cultures, GBM-RG1 and GMB12O15 (see ref.^51^) were transduced with NANEP5^+^/GFP^+^ lentivectors and co-injected at a ratio of 1:1 along with control sibling cells transduced with control/RFP^+^ vectors. All control grafts formed red intracranial tumors. However, NANEP5 did not reduce the GFP^+^ green intracranial tumor cell population (Fig. 5a–c and not shown). Given the clean results obtained above with invasive cells the reason for this outcome is unclear. Nevertheless, we first assessed *NANEP5* mRNA expression and could confirm that *NANEP5* and *GFP* mRNAs were present in transduced cells using different rt-qPCR primer pairs (Fig. 5d). Next, we examined the presence of stable protein in GBM cells transduced with NANEP5 lentivectors. Whereas FLAG-tagged NANEP5 was clearly detected in Western blots of U251 cells using anti-tag antibodies, this was not the case in the two primary GBMs tested (Fig. 5e). This result suggests that the failure of NANEP5 in these samples is due to problems with cell-type dependent translation or protein stability.

**Figure 6:**
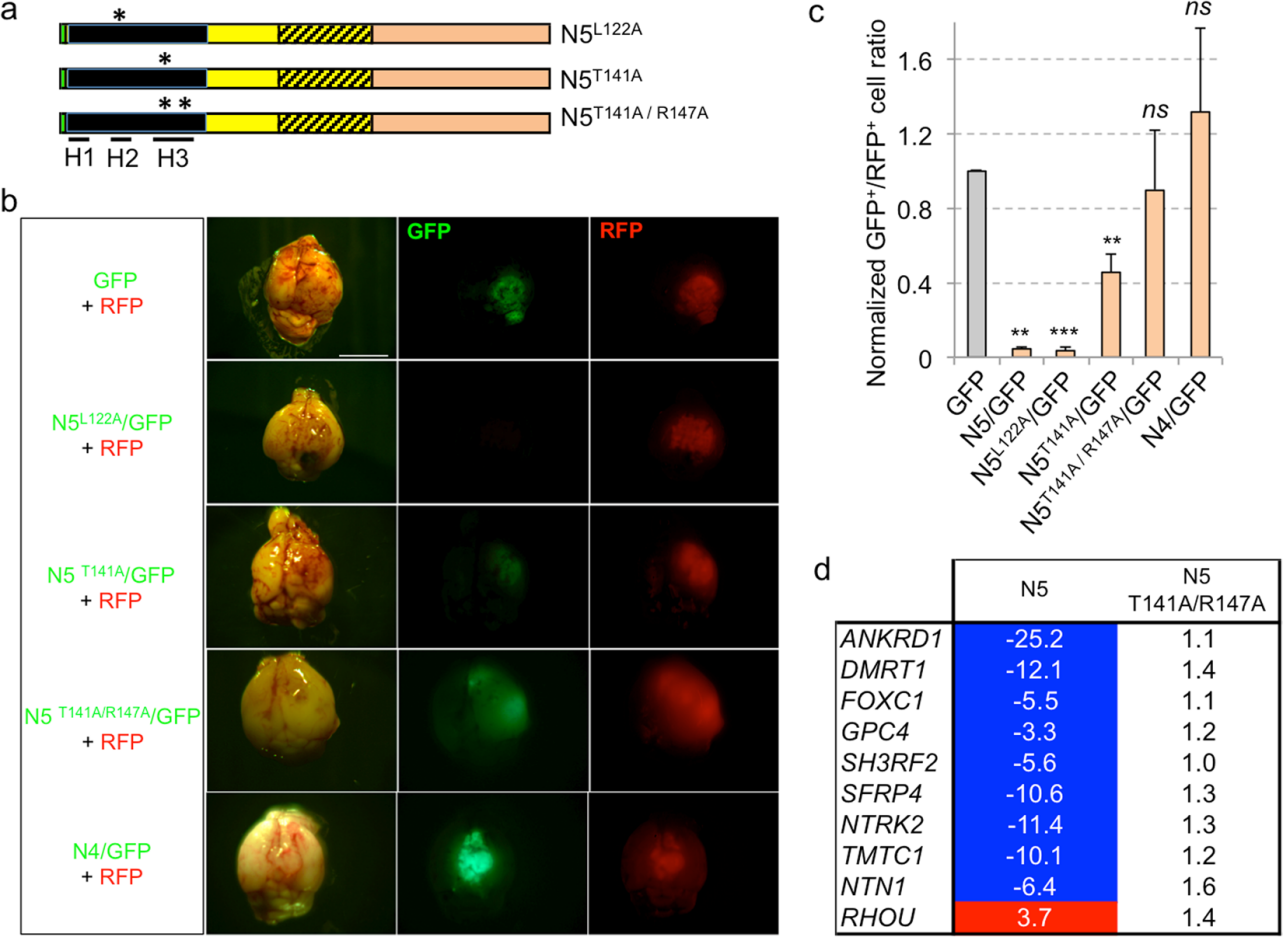
Point mutations that alter NANOG function also alter NANEP5 activity. (**a**) Box diagrams of three NANEP5 (N5) mutants (N5^L122A^, N5^T141A^, N^5T141A/R147A^) with asterisks marking position of introduced point mutations in helix 2 (H2) or helix 3 (H3) of the homeodomain. Positions of the three helices (H1-3) are underlined below the HD (black segment). (**b**) Representative images of dorsal views of dissected mice brains after orthotopic xenotransplantation of U251 red and green cells (infected with lentivectors as indicated on the left) mixed together. Pictures were taken under visible (left panels) and epifluorescent light (GFP and RFP, middle and right panels, respectively). Scale bar = 4 mm for all panels. (**c**) Quantification of the GFP^+^/RFP^+^ cells ratios in U251 tumors from (**b**) after whole tumor dissociation and FACS analysis. Ratios form control tumors were normalized to 1. Error bars are SEM. n = 16 for GFP vector alone, n = 14 for N5, n = 4 each for N5^L122A^, N^5T141A/R147A^ and N4, and n = 6 for N5^T141A^. **p < 0.05; ***p < 0.01; ns = not significative (p > 0.05). (**d**) Heat map showing the fold change (FC) in the expression of selected NANEP5 (N5) targets in NANEP5 and NANEP5^T141A/R147A^ infected cells vs. cell infected with control vectors. Negative values indicate FC of gene repression and positive values FC of gene upregulation. Blue background highlights repression at or below 2-fold. Red background: enhancement at or above 2-fold. The NANEP5^T141A/R147A^ double mutant lacks the activity of NANEP5.

Beyond context-dependent protein abundance, we sought to further test the specificity of NANEP5 action *in vivo* in U251 cells by introducing point mutations into the NHD that have been reported to alter its DNA-binding ability (Fig. 6a):

(i). L122A (using the aa counting of full-length NANOG), is located in helix 2 of the HD and enhances its DNA-binding^34^. In our assays NANEP5^L122A^ behaved as a full repressor: it mimicked NANEP5 as seen by the quantification of the number of green cells present in the brains of host mice orthotopically grafted with GFP/NANEP5^L122A^ together with control red (RFP) cells (Fig. 6b,c).
(ii). T141A is located in helix 3 of the HD has been shown to abrogate the ability of NANOG to bind the OCT4 promoter and its self-renewal function^34^. In our assays, expression of NANEP5^T141A^ resulted in a weak repressor with 5-fold less activity as compared with NANEP5 (Fig. 6b,c).
(iii). Introduction of a second DNA-binding interfering mutation, R147A also in helix 3^34^, to create the double NANEP5^T141A/R147A^ mutant, further weakened NANEP5 activity, with green cells being clearly visible in the brains of host mice: The repressor activity of NANEP5^T141A/R147A^ was not significative (Fig. 6b,c).

As controls, vector only GFP cells and GFP/NANEP4 cells were used and shown to be ineffective in reducing the number of green cells in the resulting intracranial tumors (Fig. 6b,c).

The activities of NANEP5 and the derived double point mutant were assessed on the expression of NANEP5-regulated genes obtained from RNAseq data (see below). Ten genes the expression of which was altered 2-fold or more by NANEP5 in U251 cells were chosen and tested for expression in cells transduced with NANEP5 or NANEP5^T141A/R147A^. The double mutant did not change the expression of any of these genes above or below the 2-fold threshold afforded by NANEP5, all as compared with control cells (Fig. 6d).

To extend these findings we performed luciferase reporter assays driven by co-transduced NANOG with NANEP5 or derived mutants in U251 cells. In this system, NANEP5^L122A^ and NANEP5 activities were not significantly different (Fig. S5), arguing that NANEP5 is already a strong repressor. In contrast, NANEP5^T141A^ was less active than NANEP5, and the double mutant NANEP5^T141A/R147A^ had even less repressor activity than the single NANEP5^T141A^ mutant (Fig. S5). The slight difference observed between *in vivo* red/green cell quantification and *in vitro* NBS->Luc assays may be due to the possibility that endogenous targets are more sensitive to small variations in NANEP5 function and/or that NANEP5-DNA binding affinity may depend on the exact target sequence, which is the case for NANOG^25,52^. Taken together, these results indicate that NANEP5^T141A/R147A^ is a handicapped repressor *in vivo* and *in vitro*, and suggest that the superb *in vivo* phenotype observed with NANEP5 in GBM cells in which it is made in a stable manner is due to the repression of NANOG targets.

To identify NANEP5 regulated genes, and to test if these may overlap with previously described NANOG targets, we performed RNAseq of U87 and U251 cells stably transduced with control or NANEP5 lentivectors (GSE121092). Analyses of the gene lists with FDR <0.05, 2-fold changes in expression level and CPM above log2 = 2 revealed 493 genes downregulated and 755 genes upregulated in U87; and 534 genes downregulated and 630 genes upregulated in U251 (Supplementary Excel Data Files 1 and 2).

**Figure 7:**
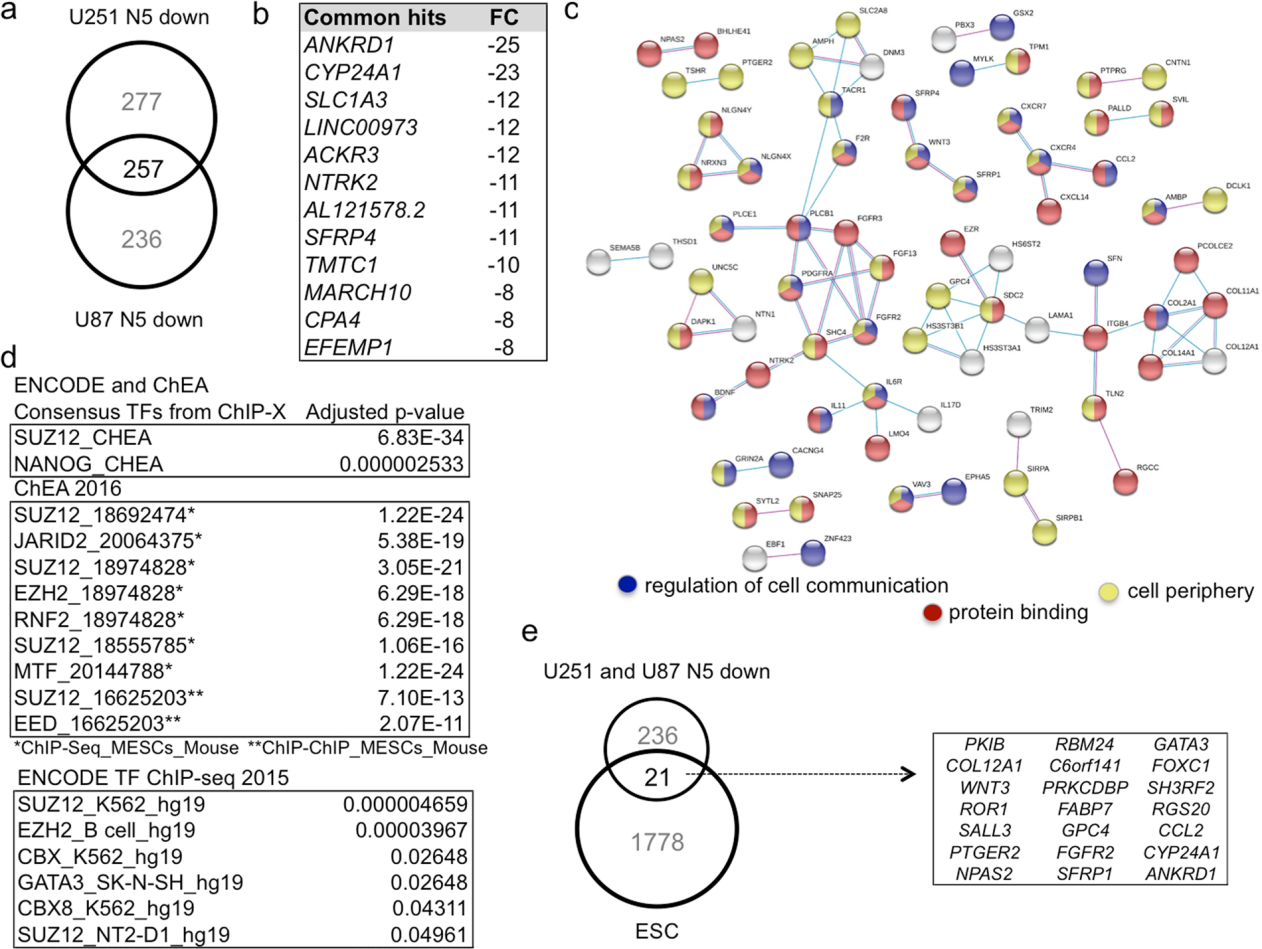
NANEP5-repressed genes include cell-cell communication components, polycomb and previously identified Nanog targets. (**a**) Venn diagram showing the number of unique and common downregulated genes in NANEP5 (N5)-expressing vs. control in U251 and U87 cells. (**b**) List of top commonly downregulated genes in NANEP5-expressing U251 and U87 cells vs. control ranked by the fold change observed in U251 cells. (**c**) STRING analysis of putative protein-protein interactions among the 257 commonly downregulated genes by NANEP5. The top GO terms for Biological Processes/Molecular Functions/Cellular Components are given in Fig. S7. Here the regulation of cell communication, protein binding and cell periphery are highlighted in blue, red and yellow, respectively, as these encompass most of the hits. The thickness of the lines denotes the strength of the interaction. (**d**) List of the top gene set enrichment analyses hits (ENCODE and ChEA, ChEA 2016, ENCODE TF ChIP-seq. 2015) of the 257 common downregulated genes by NANEP5. The adjusted P-value is given for each hit. Note the predominance of gene sets deregulated by alteration of polycomb PRC2 complex components (e.g. SUZ12, JARID2, EZH2). In ENCODE and ChEA a SUZ12 set is even more enriched in the 257 common downregulated genes than a NANOG set in ESCs. (**e**) Venn diagram showing the number of unique and shared targets among common 257 NANEP5-downregulated genes and those reported as NANOG targets by ChIP in human embryonic stem cells (ESC) by Boyer *et al*.^22^. The names of the shared 21 genes are listed in the box.

Comparing the downregulated genes in both cell types revealed only 257 common genes, representing 52% for U87 and 48% for U251 (Fig. 7a, Supplementary Excel Data File 3). Similarly, there were 353 genes upregulated in common representing 47% for U87 and 56% for U251 Supplementary Excel Data File 3). Thus, only about one half of NANEP5 regulated genes in each case are also regulated in a similar manner in the other cell type, recalling the reported context-specificity of NANOG function^26,53^.

Given the repressor nature of NANEP5, confirmed in the NBS->Luc assays above, we focused here on the 257 commonly downregulated genes and their encoded proteins (Fig. 7a; Supplementary Excel Data File 3). Transcripts upregulated by NANEP5 over control were deemed to be secondary to repressive events (Supplementary Excel Data Files 1and 2; Fig. S6). The top commonly downregulated genes (ranked by fold change in U251 cells) are shown in Fig. 7b.

Enrichment analyses of these 257 genes revealed GO Biological Processes/Molecular Functions/Cellular Components being related to cell surface receptor signaling, regulation of neural development and regulation of cell differentiation (Fig. S7). Mapping protein-protein interactions (using only the settings of experiments and databases at confidence of 0.4; https://string-db.org) uncovered several notable pairs and nodes, and most predicted interactions could be accounted for with the categories of ‘regulation of cell communication’, ‘protein binding’ and ‘cell periphery’ (Fig. 7c). These analyses highlighted several potential ligand-receptor/partner interactions: NETRIN1 with UNC5C; BDNF with NTRK2; CXCR4 with CXCL14 and CCL2; FGF13 with FGFR3 and FGFR2; IL11 and IL17D with IL6R; and WNT3 with the two secreted WNT-binding inhibitors SFRP1 and SFRP4 (Fig. 7c). Whereas most predicted interactions involved membrane and/or extracellular signaling events, the analysis also highlighted cell fate regulators and homeodomain proteins (Fig. 7c).

Interestingly, enrichment analyses of the 257 commonly repressed genes using the Enrichr software^54^ highlighted the presence of a cohort of genes previously shown to be regulated by POLYCOMB PRC2 components, notably SUZ12, JARID2, EZH2 and EED^55^ (Fig. 7d). As it has been shown that NANOG and PRC2 co-occupy many PRC2 targets in human ES cells^25,52,56^, our data suggested the possibility that NANEP5 might interact with PRC2 components to regulate target genes in cancer cells. Indeed, 58 of the 257 genes (22.5%) are also SUZ12 targets in ESCs^56^ (Fig. S8) and include the CXCR4-CXCL14, NETRIN1-UNC5C, WNT3-SFRP1/4, BDNF-NTRK2 and FGF13-PGFRA-PLCB1 signaling nodes and axes. If and how NANEP5 and PCR2 components interact remains unclear since our co-immunoprecipitation analyses with tagged NANEP5 and PRC2 components failed to reveal stable associations (Fig. S9 and not shown).

Finally, we found that 21 of the 257 downregulated genes (8%) were previously identified by chromatin immunoprecipitation in hESC as bona fide NANOG targets^25^ (Fig. 7e). Several of these 21 encoded proteins participate in intercellular signaling such as the CCL2 ligand; the FGF receptor 2; the WNT3 ligand as well as GPC4, which can act as a WNT signaling regulator^57–59^, and SFRP1, a secreted WNT inhibitor. Notably, the set also includes genes encoding transcription factors reported to regulate stemness in different contexts: *SALL3*, *GATA3* and *FOXC1*^60–62^.

## Discussion

Whereas a number of current therapeutic strategies aim to target cancer stem cells^63,64^, we chose to block NANOG function in human GBM since it is a key transcription factor that specifically regulates stemness in multiple contexts^18,25,27^. NANOG acts downstream of signaling inputs that might show redundancy^65^, and is largely absent from normal adult human tissues (https://www.proteinatlas.org/ENSG00000111704-NANOG/tissue)^6^. Beyond RNA interference^14,18,31^ it has proven difficult to target transcription factors with small molecules or antibodies due in part to the difficulty of blocking protein-DNA contacts and the poor accessibility of circulating antibodies to the cell nucleus^66,67^. Given the present inability to effectively treat metastases – thought to derive from colonizing cancer stem cells- and the rapid progression of invasive cancers such as GBM, unconventional strategies need to be investigated. We have thus explored the possibility that chimeric repressors, widely used in developmental studies, could be useful as potential anti-cancer agents targeting stemness, focusing in this study on NANOG.

Here we provide a proof of principle that chimeric NANOG repressor proteins, named NANEPs, could be used as anti-tumor molecules in a context-dependent manner. For most of the NANEP development part we opted to use two different GBM cell lines that allow ease of transduction, culture and have different properties. U87 are not very invasive but U251 are highly invasive^49^ and the latter thus provide a good model for tumor cell penetration into and invasion of the brain parenchyma after orthotopic grafting.

Given the known activities of the domains used - HD for DNA-binding^45,68^, the WR dimerization domain for the maintenance of pluripotency^36^, and the HES1 repressor sequences as a transposable, Gro/TLE-binding repressor domain^46^ - as well as the altering affects of the point mutations introduced^34^ we conclude that NANEPs very likely act by blocking endogenous NANOG function. Indeed, NANEP5 mimics the reported activities of NANOG kd *in vitro* and *in vivo*^18^ providing confidence in our approach since only about 50% of downregulated genes in U87 or in U251 are also downregulated in the other (see above) due to the large context-dependency of NANEP/NANOG function.

The present results with NANEP5 therefore raise the possibility that the activating function of NANOG is critical for its role in tumor stemness and growth. However, whether NANEPs can directly inform on the function of NANOG *in vivo* remains to be clarified as no meaningful *in vivo* transcriptome could be determined since NANEP5 abolishes all growth of intracranially implanted U251 cells. In addition, NANEP5 is a chimeric dominant repressor protein that does not likely have the same kinetics, binding partners and affinities than NANOG, and thus NANEP5 and NANOG regulated genes may not be entirely overlapping. We have also used the reported binding of Nanog to gene-associated regions in global chromatin immunoprecipitation analyses in ES cells^25^ as a guide to highlight genes that could be both NANEP- and NANOG-regulated. In doing so, we highlight 21 genes that include *FOXC1*, which has been reported to be important for GBM growth and invasiveness^69,70^.

The analyses of NANEP5 repressed genes raise possible explanations for its superb effects. Although the RNAseq was necessarily done *in vitro*, we interpret the changes as affecting functions required *in vivo* and in clonogenic assays but not for *in vitro* expansion in standard 2D conditions with serum. Thus, we can investigate the changes observed with and without NANEP5 and try to relate them to the *in vivo* phenotypes. In this light, it is interesting that there is an accent on cell-signaling/membrane/periphery events and in those at play in axon guidance. The combined effects of repressing intercellular communication (e.g. aspects of NETRIN, BDNF, PDGF, NOTCH (DLD1), WNT and FGF signaling), as well as compromising the function of key transcription factors involved in stemness in different contexts (e.g. IRXs, PBX3, GSX2, SALL3, FOXC1, GATA3, etc.) would seem likely sufficient to inhibit tumor growth. Interestingly, ligand-receptor interactions are druggable and blocking two or more of those highlighted here could yield strong synergistic anti-tumor effects. For instance, we note that *FOXC1*, *NETRIN1* and its receptor UNC5C are coherently repressed by NANEP5 and that NETRIN signaling and FOXC1 have been shown to be required for GBM growth^69–71^. Indeed, these targets could, in principle, already account for the strong NANEP effects.

Enrichment analyses on the commonly NANEP5-repressed genes raised the possibility that NANEP5 could interact or affect polycomb PRC2 complex function given the overlap of NANEP5-regulated genes with those regulated by SUZ12 and other PRC2 components. This hypothesis is attractive given that NANOG and NANEP5 functions appear to be context-dependent, that NANOG can influence and is influenced by the epigenetic states of cells^72,73^, and that in ES cells about one third of NANOG-targets overlap with PRC2-occupied genes encoding developmental transcription factors^25,56^. However, our co-IP results in human U251 GBM cells did not reveal stable binding. Whether or how NANEPs and PRC2 interact thus remains to be clarified.

More generally, the comparison of the kinds of downregulated and upregulated genes suggest that NANEP5 drives a profound remodeling of the cell, that include transcriptional, membrane and ligand properties, which appear to be incompatible with cancer growth and persistence.

The present data suggests that NANEPs could be further developed for their use following debulking surgery or in combination with chemo- and radiation therapies. To this end, several issues need to be addressed before such a strategy can be realized. The first potential difficulty may stem from the nature of the approach: a chimeric protein may be recognized by the immune system and repeated doses may not be tolerated and/or effective. One goal in this sense would be to engineer the smallest possible NANEPs with the highest activity, although all NANEPs will likely be large in comparison to most hormones since the NHD alone is already 60aa-long. However, even if NANEPs only extend the initial survival period from diagnosis after current treatments (between 11 and 31 months depending on *IDH* and *MGMT* status: https://www.abta.org/tumor_types/glioblastoma-gbm/), this would be an important improvement. As anti-stemness agents, however, NANEPs could well have additional and beneficial long-term effects following short treatments.

A second issue derives from the observed differential translation/stability of NANEP5 in different cells. Notably, the lack of stable protein in the primary GBMs tested suggests that further research is needed to find ways to enhance the production of stable protein or that patient tumors may need to be screened to find those that allow NANEP production. NANOG stability, and by extension possibly that of NANEPs, is regulated as suggested by various findings, including i- aspirin treatment decreases the levels of stable NANOG protein in cycloheximide-treated cancer cells^33^, ii- PIN1 enhances the stability of phosphorylated NANOG^74^, iii- SRSF3 promotes NANOG mRNA nuclear export and thus protein production^75^, iv- the deubiquitinase USP21 stabilizes NANOG^76^.

Stable NANEP5 protein is expected to require TLE co-repressors for function given the presence of the HES1 repressor domain^46^. It is thus notable that *TLE2* is upregulated in U251 NANEP5-expressing cells (Fig. S6. Supplementary Excel Data File 3). This suggests that a byproduct of stable NANEP5 function may be its increased activity by enhanced levels of repressor co-factors, possibly supporting its high potency in the ‘off’ state (without DOX) of the conditional system we have used. Whether this loop exists in other cancer cells remains to be determined but it suggests a high repressor efficiency of NANEP5 when this is stable.

A third item to design is the delivery method. Two ways can in principle be envisioned to deliver NANEPs to tumor cells in patients: either via viral vector transduction (e.g. using lentiviruses) or by delivering mature protein. In the first case, clinical trials are currently being performed with different viral vectors to deliver different cargoes^77,78^ and delivering NANEPs should, in principle, not be different. Here it would be necessary to explore the best vectors to use and the delivery mode, perhaps bathing the surgery margins with mature viral particles aiming to infect cancer stem cells left behind that may be responsible for residual disease. As for protein delivery, several additional hurdles may need to be resolved, including cost-effective scaling-up and modification of NANEP to become a transducing protein, possibly via fusion with cell penetrating peptides or with tumor homing peptides such as transferrin^79,80^.

In summary, whereas additional work is required before the clinical use of NANEPs, the results presented here provide the basis for their development as effective yet context-dependent anti-tumor agents.

## Methods

### Cell culture

U251 and U87 cells were purchased from ATCC and cultured following standard protocols with 10% fetal calf serum. Primary GBM cells were cultured as spheroids in Neurobasal media (*Thermo Fisher*) supplemented with B27 (1:50) (*Invitrogen*), GlutaMAX (1:100) (*Invitrogen*), penicillin/streptomycin (1:100), 0.1% heparin (*Sigma*), 10 ng/mL EGF and 10 ng/mL FGF2. GBM-RG1 and GBM-12O15 were from ref.^51^ and originally obtained by dissociation of human GBM surgical specimens after patient´s written informed consent from all patients and ethical approval (reviewed and approved by the Research Ethics and Animal Welfare Committee at the Instituto de Salud Carlos III, Madrid, Spain)^51^. All methods were performed in accordance with the relevant guidelines and regulations. For the conditional DOX-inducible NANEP system DOX (*Pelodis*) was added to complete media *in vitro* to a final concentration 1 *μ*g/ml.

### Animal experimentation

Mice were kept in SPF animal facility and monitored for well being at a daily basis. All *in vivo* experiments involved NMRI NUDE mice (*Janvier*). All experiments, including those involving human cells, were performed in accordance with relevant guidelines and regulations, including animal procedures performed under approved protocols of the Office Cantonal Vétérinaire de Genève, Switzerland (GE-77-17).

### Plasmids and lentiviral vectors

All NANEPs and NHD1-3 were designed in the lab. All, except N01 and N02, were codon-optimized and cloned into *Evitria’s* EF1-expression vectors. N01 and N02 were cloned in-house into a pCMV expression vector. Lentiviral vectors used in the study were pRRL-CMV-PGK-GFP/RFP-WPRE (pTWEEN) (see ref.^18^). NANEPs expression is driven in this vector from CMV promoter, whereas GFP is driven by a PGK promoter. Control empty lentivectors express only GFP or RFP. For the conditional DOX-inducible NANEP5 expression system, a lentivector encoding rtTA was used allowing DOX induction, as described before^27^.

### Luciferase assays

293T cells were transfected with calcium phosphate using standard protocols. Each transfection reaction combined a HSV-TK Renilla plasmid as a control and 3x NANOG-Binding Site (NBS) luciferase reporter cloned into pWPI vector. Cells were co-transfected with vectors expressing NANOG and different NHDs or NANPEs. 24 to 36 h after transfection cells were lysed with passive lysis buffer (*Promega*) and subjected to a single freeze–thaw cycle at −80 °C. Luciferase activity was measured for each reaction by mixing cell lysate with Luciferase Assay Buffer (*Promega*) in white 96-well plates using VICTOR^3^ multilabel counter (*PerkinElmer*).

### RNA extraction, RT-PCR and quantitative RT-PCR (qRT-PCR)

Total RNA was extracted using TRIzol Reagent (Ambion) following the manufacturer’s protocol. 2 μg of RNA was used for cDNA synthesis in total volume of 20 μl (SuperScriptII, *Invitrogen*). cDNA reactions without reverse transcriptase were used as negative controls. For RT-PCR 2 μl of cDNA synthesis reaction were next used for PCR using GoTaq polymerase (*Promega*) and specific primers. For qRT-PCR, the cDNA synthesis reaction was diluted 25x and 5 μl were used for each qPCR reaction (iQ^TM^ SYBR Green Supermix, *BIO-RAD*).

Primers used were as follows:

*ANKRD1:*

*fwd AGTAGAGGAACTGGTCACTGG*,

*rev TGTTTCTCGCTTTTCCACTGTT;*

*NTN1:*

*fwd TGCAAGCCCTTCCACTACG*,

*rev TGTTGTGGCGACAGTTGAGG;*

*FOXC1:*

*fwd TGTTCGAGTCACAGAGGATCG*,

*rev ACAGTCGTAGACGAAAGCTCC;*

*GFP:*

*fwd GTAAACGGCCACAAGTTCAG*,

*rev GAAGTCGTGCTGCTTCATGT;*

*NANEP5 pair 2 from* Fig. 5*:*

*fwd AAGCAGAAGACCAGGACAGTG*,

*rev CGTTGGGTTGACCAGACAGCC;*

*NANEP5 pair 3 from* Fig. 5*:*

*fwd AATATTCAGTCATGGTCCAAC*,

*rev AATCACGGGACCACTGTGAGC;*

*NTRK2:*

*fwd ACCCGAAACAAACTGACGAGT*,

*rev AGCATGTAAATGGATTGCCCA;*

*RHOU:*

*fwd GCTACCCCACCGAGTACATC*,

*rev GGCTCACGACACTGAAGCA;*

*SFRP4:*

*fwd CCTGGAACATCACGCGGAT*,

*rev CGGCTTGATAGGGTCGTGC;*

*TMTC1:*

*fwd ACGGTGTCTCCCTTCTTCTTG*,

*rev ATTGCTCGACTTGTCTTGCTT;*

*DMRT1:*

*fwd CAGAGGGACGTATGGTCATCC*,

*rev GCGGGCAGTTGTATAGATTGTTG;*

*GPC4:*

*fwd GTCAGCGAACAGTGCAATCAT*,

*rev ACATTTCCCACCACGTAGTAAC;*

*SH3RF2:*

*fwd GGACGCCTGTGTTTTCCAAC*,

*rev TGAGCGCACTCCATCCAGA*.

### RNA sequencing and analyses

RNA was extracted as described above. Each condition was independently processed in triplicates and the libraries were prepares using Illimina TruSeq protocols. The samples were sequenced on a HiSeq Illumina 4000 machine with 50 bp reads followed by standard analyses. Transcripts represented only by 2 reads or less in both compared conditions were eliminated.

### Collagen invasion assay

500 U251 cells were resuspended in 20 μl of media and cultured for 48 h in a hanging drop. Aggregates of cells were next placed onto a collagen I (*Gibco*) cushion and covered with EMEM media. Cell invasion was allowed to occur for next 48 h and invasive spread was analyzed under the light microscope.

### Co-Immunoprecipitation

293T cells were transfected with calcium phosphate using standard protocols. 36 h after transfection, proteins were extracted using IP lysis buffer (50 mM Tris, 150 mM NaCl, 2 mM EDTA, 0.5% NP40, 8.7% glycerol) supplemented with a fresh protease inhibitors cocktail (cOmplete^TM^ MINI, EDTA free, ref: 11836170001). Approximately 2.5 mg of proteins were used for each immunoprecipitation reaction. Protein lysates were first pre-cleared with Protein A/G PLUS-agarose beads (*Santa Cruz Biotechnology*) for 30 minutes. Next, they were incubated with primary antibodies (anti-flag antibody: F7425 from *Sigma*, anti-HA antibody C29F4 from *Cell Signaling*) for 2–6 h at 4 °C and next with Protein A/G PLUS-agarose beads either for 2 h or overnight at 4 °C. Protein-beads complexes were next washed following manufacturer’s protocol, resuspended in 5x Laemmli buffer (62.5 mM Tris-HCl, pH 6.8 25% glycerol, 2% SDS, 0.01% Bromophenol Blue) supplemented with 10 mM DTT, boiled for 5 minutes at 95 °C, fast-frozen and stored at −80 °C until SDS-PAGE.

### Protein expression and Western blot analysis

Cells transfected or infected with control or NANEP-expressing vectors were lysed with RIPA buffer (50 mM Tris pH 7.4, 1% NP40, 0.25% sodium deoxycholate, 150 mM NaCl, 1 mM EDTA) supplemented with fresh protease inhibitors for 20 minutes on ice. Proteins were then analyzed on SDS-PAGE electrophoresis followed by Western Blot using specific primary antibodies anti-flag antibody: F3165 from *Sigma* (1:1000), anti-HA antibody C29F4 from *Cell Signaling* (1:1000), anti-GAPDH: 2118S from *Cell Signaling* (1:1000), GFP NB1001770 from *Novusbio* (1:1000) and appropriate HRP secondary antibodies (HRP anti mouse 170-65-16 (1:3000), HRP anti rabbit 170-6515 (1:3000), and HRP anti-goat 1721034 (1:5000) from *BioRad*). Chemiluminescence was determined using the ECL detection kit (*GE Healthcare Amersham*) and band intensities were quantified using Image Studio Lite software.

### Immunofluorescence

U87 cells were transfected with Lipofectamine 2000 using standard protocols. 36 h after transfections they were fixed with fresh 4% PFA pH8 and subjected to primary antibody (mouse anti-flag F3165 from *Sigma* (1:1000) overnight at 4 °C and then secondary antibody stainings (ref: 2118S from *Life Technologies*) for 1 h at room temperature. Nuclei were stained with DAPI (1:10000) for 2 minutes at rt. Pictures were taken using a Confocal Line scan LSM 800 Airyscan microscope at the Imaging Platform of the University of Geneva School of Medicine.

### Transwell assay

1 × 10^6^ U251 LacZ^+^ cells were plated in 100 mm dish in DMEM/F12/10%FBS. The day after media was changed for DMEM/F12/0.5%FBS and cells were cultured in these conditioned for the following 16 hours. Next, cells were tripsinized, washed with PBS 3x and 50’000 of cells were plated in 100 μl of DMEM/F12/0.5%FBS onto the transwell (ref: 3422 from *Sigma*) with 600 μl of the same media but containing 10%FBS in the bottom chamber. Migration was allowed for 3–6 hours. Cells were then fixed with fresh 4% PFA and stained with X-Gal buffer and substrate for 1 hour at 37 °C.

### CD133^+^ cells isolation and spheroid cultures

10 days after infection, U251 cells were collected and dissociated into a single cell suspension using Stem Pro Accutase (*Thermo Fisher*). CD133^+^ cells were next isolated using a MACS separation kit (*Miltenyi Biotec*) following the manufacturer’s protocol and counted under a light microscope. 1000 (for ‘clonal conditions’ or 10000 (for ‘bulk conditions’) CD133^+^ cells were next plated in 6 ml of 3D culture media (DMEMF12 media, 1xP/S, 10 ng/ml EGF, 10 ng/ml FGF, 1x B27 supplement (Gibco)) in duplicates or triplicates and cultured for 4 weeks. 1 ml of fresh 3D culture media was added to the flasks every 10 days. Pictures of the spheroids were taken on the 21^st^ day of 3D culture. The entire experiment was independently repeated 3 times.

### Red/green *in vitro* assay

U251 cells were infected with RFP, GFP and N5/GFP expressing lentivectors separately. GFP^+^ and N5/GFP^+^ cells were then mixed with RFP^+^ cells at ratio 1:1 and plated. At days 4 and 8 of co-culture, cells were trypsinized, washed and analyzed by FACS.

### Subcutaneous xenografts

U251 and U87 cells were infected with control GFP or N5/GFP expressing lentivectors at MOI = 4–5. 10 days after infection cells were trypsinized and resuspended at concentration of 5 × 10^5^ cells/100 µl HBSS. 100ul of cells suspension was used for each injection site: into 2 flanks and on the back of each mouse. For red/green subcutaneous xenografts, GFP^+^ and N5/GFP^+^ cells were mixed with sibling RFP^+^ cells at ratio 1:1 (confirmed by FACS) and injected as described above. The resulting tumors were allowed to grow for couple of weeks and taken before reaching the local legal limit. Subcutaneous tumors were measured, weighted and pictured under visible and fluorescent light using a Lightools Research CCD camera. Next, they were dissociated as described in ref.^27^, and analyzed by FACS.

### Red/green orthotopic xenografts

Grafts were performed following the protocol in ref.^18^: GBM cells were infected as above and 3 × 10^5^ dissociated cells were resuspended in 4–5 μl of HBSS and injected intracranially at coordinates {x = 1, y = −2, z = −2.5 for U251; x = −1, y = 1, z = −2.5 for U87 and x = 2, y = 0.5, z = −3 for primary GBM RG1, and 12O15 cells} relative to the bregma point using a stereotaxic frame. Mice were sacrificed immediately after occurrence of the first signs of pain or neurological disease. Whole mice brains were dissected and pictured under visible and fluorescent light. Tumors were dissociated and cells were FACS analyzed.

For confocal imaging of the intracranial U251 red/green tumors, mice were anesthetized and perfused with fresh 4%PFA using a peristalitic pump. Their brains were next dissected, soaked in fresh 4%PFA overnight and washed with 1xPBS. Coronal sections using a Leica (VT1000S) vibratome were performed on whole brains and checked under the microscope for the presence of fluorescent tumor cells. Brain slices (of 30 μm thickness) containing tumors were next analyzed and pictured by confocal microscopy as above.

A patent application covering NANEPs has been granted to the University of Geneva (WO/2012/176175).

## Supplementary information

Supplementary information

Supplementary Excel Data File 1

Supplementary Excel Data File 2

Supplementary Excel Data File 3
