# Supplementary material for: Chimeric NANOG repressors inhibit glioblastoma growth in vivo in a context-dependent manner
Source: Sci Rep. 2019 Mar 7;9:3891. doi: 10.1038/s41598-019-39473-y (PMC6405761; doi:10.1038/s41598-019-39473-y)
Supplement: Supplementary file 1 — Supplementary information [file 41598_2019_39473_MOESM1_ESM.pdf]

Chimeric NANOG repressors inhibit glioblastoma growth in vivo in a context-dependent manner

Supplementary Figues

Monika Kuciak[1], Christophe Mas[1,&], Isabel Borges[1], Pilar Sánchez-Gómez[2] and Ariel Ruiz i Altaba[1]*

[1]Department of Genetic Medicine and Development
University of Geneva Medical School
Rue Miquel Servet 1CH-1211 Geneva, Switzerland
[2]Neuro-oncology Unit, Instituto de Salud C.III-UFIEC, Madrid, Spain

[&]Present address: Oncotheis Sàrl. 18 chemin des Aulx,
CH-1228 Plan-Les-Ouates, Geneva, Switzerland

*Corresponding author: Ariel.RuizAltaba@unige.ch; 41-22-379-5646

Supplementary figures

Figure S1. NANOG binding site-*luciferase* reporter plasmid.

a) Scheme of NANOG binding site (NBS) reporter plasmid built and used in this study. Sequences corresponding to the region marked with dashed lines are shown above the scheme. Underlined sequences correspond to BamHI restriction sites used for cloning three copies of a NBS fragment. This resulted from the hybridization of sense and antisense oligonucleotides for the sequence 5'-GATCCGCTAGCACCCTTCGCCGATTAAGTACTTAAG-3' introduced into the pGL3 Luciferase reporter vector (Promega) by BamHI ligation. An NBS-Luc PCR amplicon amplified from this vector with the following primers 5'-ATGCCTCGAGACTAGTCTTATCATGTCTGGATCC-3'; 3'-AGTCCTCGAGCGTCATCGCTGAATACA-5' was then cloned XhoI into the pWPI backbone to generate a NANOG Luciferase reporter lentivector. The actual NBS is shown in red. The 5 'end of the *luciferase* gene is in green.

b) Validation of the NBS reporter. Dual Firefly/Renilla luciferase assays were run and relative light units (RLU) quantified in 293T cells testing for the activities of NANOG, as well as NANOG plus NANEP01 or NANEP02 (N01 or N02). The NBS alone (first bar) reports background activity. Signal in NBS + NANOG was used as control to calculate *P values* for all conditions. Error bars are SEMs. ** = $p<0.05$.

c) Quantification of the Western blot result shown in Fig. 1d. Protein expression is represented as a FLAG-tagged construct/GAPDH signal ratio for each sample.

Figure S2. NHD and NANEP sequences.

Amino acid sequences of NHDs 1-3 and NANEPs 01-11 used in this study. Colors as annotated at the top of the figure as follows: blue: strep and flag tags; yellow: NANOG sequences with the homeodomain in bold and the WR dimerization domain underlined; green: linkers; pink: repressors with minimal active domains in pink and bold.

Figure S3. Activities of lentiviral NANEP constructs.

Quantification of NANOG binding site (NBS) reporter activity in dual Firefly/Renilla luciferase assays testing NANEP4 and NANEP5 lentiviral constructs. The histogram shows NBS relative light units (RLU) in 293T cells driven by NANOG and its repression by co-expressed NANEP4 or NANEP5 from transduced vectors. Signal in NBS (first bar) represents background activity. The level of activity from NBS + NANOG was used to calculate *P values* for all other conditions. Error bars are SEMs. ** = p<0.05.

Figure S4. Leaky low expression of NANEP5 from a DOX-inducible conditional expression system is enough to inhibit tumor growth from expressing cells.

a) Normalized *NANEP5* mRNA (*N5*) expression levels in control U251 cells transduced with the constructs described with and without doxycycline (DOX). Individual values are given above each bar. Note that in U251 rtTA cells N5 is not detected, as expected. However, it is present in the N5 conditional system without DOX induction, although at much (~56 fold) lower level than when compared to straight N5 expression. DOX treatment (+DOX) induces N5 levels 100-fold over those detected in the -DOX condition.

b) Experimental approach of orthotopic brain transplantation of U251 rtTA cells infected with RFP mixed at a 1:1 ratio with U251 cells expressing NANEP5^{conditional}/GFP. The lower portion shows representative images of dorsal views of dissected mouse brains one month after orthotopic injections and without DOX administration. Note that without DOX induction U251 rtTA/RFP + NANEP5^{conditional}/GFP cells formed a brain tumor but this was not green (GFP^{+}), indicating that residual NANEP5 function obliterated expressing cells. Pictures were taken under visible (left panels) and fluorescent light (GFP and RFP, middle and right panels, respectively).

Scale bar=3.5mm.

Figure S5. Activities of NANEP5 point mutants.

Histogram of dual luciferase activities in 293T cells testing the function of three NANEP5 mutants - $N5^{L122A}$, $N5^{T141A}$ and $N5^{T141A/R147A}$ - on the repression of NANOG-driven signal. NBS activity is given in relative light units (RLU). Signal in NBS (first bar) represents background activity. Stars (** = $p<0.05$) indicate *P values* for all conditions, that were calculated using signal in NBS + NANOG as a control. *P values* are also given for all N5 mutants vs. N5 as noted. Error bars are SEMs.

Figure S6. NANEP5 upregulated genes.

a) Venn diagram showing the number of unique and common upregulated genes in NANEP5 (N5)-expressing vs. control U251 and U87 GBM cells.

b) Top 10 common upregulated genes by NANEP5 vs. control in U87 and U251 GBM cells ranked by the fold change (FC) in U251 cells. See Supplementray Excel data file 1 and 2 for the full list.

c) STRING protein interaction analyses highlighted top Biological Process GO terms of defense and inflammatory responses, as well as GO Cellular Component terms of Extracellular Region and Plasma Membrane including extracellular matrix, signaling and receptor components, which are shown in the map. The thickness of the lines denotes the strength of the interaction. In addition to the analyses in (d) we highlight the upregulation of TLE2 and HES1, with the former being a potential co-repressor partner of NANEP5.

d) Top gene sets from enrichment analyses with the 353 common upregulated genes by NANEP5. The adjusted P-value is given for each one. Enrichment analyses highlights include cytokine (interleukin, interferon) signaling, leukotriene D4 biosynthetic and ketosteroid monooxygenase activity networks.

Figure S7. Top GO enrichment terms of the common NANEP5-downregulated gene set.

The top GO terms for Biological Processes/Molecular Functions/Cellular Components resulting from String enrichment analyses of 257 commonly downregulated genes by NANEP5 in U251 and U87 cells.

Figure S8. Identity of the 58 genes in common between the NANEP5-downregualted set and POLYCOMB SUZ12 targets in ESCs.

Venn diagram showing the number of unique and shared targets among common 257 NANEP5-downregulated genes and those reported as SUZ12 targets by genome wide ChIP-ChIP in human embryonic stem cells (ESC) by Lee et al., (2005). The names of the shared 58 genes are listed in the box.

Figure S9. NANEP5 does not associate in a stable manner with selected PRC2 components.

Western blots with anti-HA and anti-Flag tag antibodies after immunoprecipitation with anti-HA antibody (IP^{HA}). 293T cells were transfected with NANEP5 (Flag-N5) and different HA-tagged PRC2 proteins (HA-EZH2, HA-EED, HA-SUZ12, HA-JARID2), as annotated above the pictures. Flag-N5 was not detected in any of the IP^{HA} fractions. IN: input.

Supplementary Excel Data File 1.

List of significantly upregulated and downregulated genes with fold change of equal or greater than 2. Results shown are for U87 cells with NANEP5 compared with sibling U87 cells transduced with vector alone controls.

Supplementary Excel Data File 2.

List of significantly upregulated and downregulated genes with fold change equal or greater than 2. Results shown are for U251 cells expressing NANEP5 compared with sibling U251 cells transduced with vector alone controls

Supplementary Excel Data File 3.

List of commonly upregulated and downregulated genes in both U87 and U251 cells transduced with NANEP5 as compared individually with appropriate controls.

a

5′TCTTATCATGTCTGGATCCGCTAGCACCCTT**CGCCGATTAAG**TACTTAAGGATCCGCTAGCACCCTT**CGCCGATTAAG**TACTTAAGG
ATCCGCTAGCACCCTT**CGCCGATTAAG**TACTTAAGGATCCTCTAGAGTCGACCTGCAGGGCCAGCCCAGGCTCCGGGGCACGTAAAAGC
GGGGCTGTGAGACCGGAGAGCACGGAGCGACCAGCCAGGGCTGAGCTGNGGAGACGGTGAGCAGGGCTGTGCGGGCAGCCAAGCTT GG
CATTCCGGTACTGTTGGTAAAATG**GAAGACGCCAAAAACATAAAGAAAGGCCCGGCGCCATTCTATCCTCTAGAGGATGGAACCGCTGG**
AGAGCAACTGCATAAGG-3′

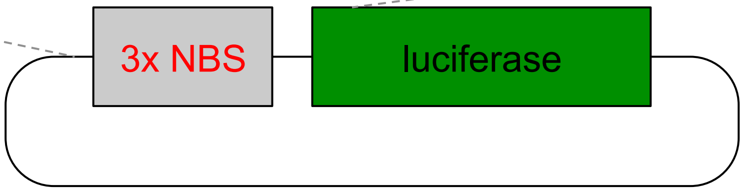

b

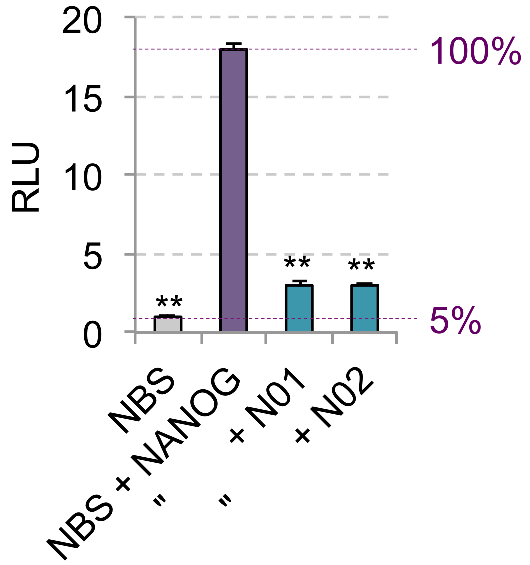

c

	Flag-tagged construct/GAPDH
NHD1	0.1
NHD2	1.3
NHD3	0.3
N4	3.2
N5	0.9
N6	1.5
N7	0.9
N8	1.5
N9	0.6
N10	0.5
N11	0.2

Figure S1. Kuciak et al.

Figure S2 (i). Kuciak et al.

Strep and Flag Tags
NANOG sequences: **HD in bold**, WR underlined
Linkers
Repressors (active domains **in bold**)

NHD1

MWSHPQFEKASDYKDDDDKLAAAVPVK**KQKTRTVFSSTQLCVLNDRFQRQKYLSLQQMQELSNILNLSYKQVKTWFQ**
NQRMKSKRWQ

NHD2

MWSHPQFEKASDYKDDDDKLAAAVPVK**KQKTRTVFSSTQLCVLNDRFQRQKYLSLQQMQELSNILNLSYKQVKTWFQ**
NQRMKSKRWQKNNWPKNSNGVTQKASAPTYPSLYSSYHQ

NHD3

MWSHPQFEKASDYKDDDDKLAAAVPVK**KQKTRTVFSSTQLCVLNDRFQRQKYLSLQQMQELSNILNLSYKQVKTWFQ**
NQRMKSKRWQKNNWPKNSNGVTQKASAPTYPSLYSSYHQGCLVNPTGNLPMWSNQTWNNSTWSNQTQNIQSWSNHSW
NTQTWCTQSWNNQAWNSPF

NANEP01

MDYKDDDDKLAAAVPVK**KQKTRTVFSSTQLCVLNDRFQRQKYLSLQQMQELSNILNLSYKQVKTWFQNQRMKSKRWQ**
KNNWPKNSNGVTQKASAPTYPSLYSSYHQGSTMALEDRCSPQSAPSPITLQMQHLHHQQQQQQQQQQMQHLHQLQQ
LQQLHQQQLAAGVFHHPAMAFDAAAAAAAAAAAAAAHAHAAALQQRLSGSGSPASCSTPASSTPLTIKEEESDSVIG
DMSFHNQTHTTNEEEEAEEDDDIDVDVDDTSAGGRLPPPAHQQQSTAKPSLAFSISNILSDRFGDVQKPGKSMENQA
SIFRPFEASRSQTATPSAFTRVDLLEFSRQQQAAAAAATAAMLERANFLNCFNPAAYPRIHEEIVQSRLRRSAANA
VIPPPMSSKMSDANPEKSALGSRVASL

NANEP02

MDYKDDDDKLAAAVPVK**KQKTRTVFSSTQLCVLNDRFQRQKYLSLQQMQELSNILNLSYKQVKTWFQNQRMKSKRWQ**
KNNWPKNSNGVTQKASAPTYPSLYSSYHQGCLVNPTGNLPMWSNQTWNNSTWSNQTQNIQSWSNHSWNTQTWCTQSW
NNQAWNSPFSTMALEDRCSPQSAPSPITLQMQHLHHQQQQQQQQQQMQHLHQLQQLQQLHQQQLAAGVFHHPAMAF
DAAAAAAAAAAAAAAHAHAAALQQRLSGSGSPASCSTPASSTPLTIKEEESDSVIGDMSFHNQTHTTNEEEEAEEDD
DIDVDVDDTSAGGRLPPPAHQQQSTAKPSLAFSISNILSDRFGDVQKPGKSMENQASIFRPFEASRSQTATPSAFTR
VDLLEFSRQQQAAAAAATAAMLERANFLNCFNPAAYPRIHEEIVQSRLRRSAANAVIPPPMSSKMSDANPEKSALG
SRVASL

NANEP4

MWSHPQFEKASDYKDDDDKLAAAVPVK**KQKTRTVFSSTQLCVLNDRFQRQKYLSLQQMQELSNILNLSYKQVKTWFQ**
NQRMKSKRWQKNNWPKNSNGVTQGVAPGASPGGAAPPPGGAPCKLGSQAGEAAKVFGGFQVVPAPDGQFAFLIPNGA
FAHSGPVIPVYTSNSGTSVGPNAVSPSSGPSLTADSM**WRPW**

NANEP5

MWSHPQFEKASDYKDDDDKLAAAVPVK**KQKTRTVFSSTQLCVLNDRFQRQKYLSLQQMQELSNILNLSYKQVKTWFQ**
NQRMKSKRWQKNNWPKNSNGVTQKASAPTYPSLYSSYHQGCLVNPTGNLPMWSNQTWNNSTWSNQTQNIQSWSNHSW
NTQTWCTQSWNNQAWNSPFPGGAAPPPGGAPCKLGSQAGEAAKVFGGFQVVPAPDGQFAFLIPNGAFAHSGPVIPVY
TSNSGTSVGPNAVSPSSGPSLTADSM**WRPW**

NANEP6

MWSHPQFEKASDYKDDDDKLAAAVPVK**KQKTRTVFSSTQLCVLNDRFQRQKYLSLQQMQELSNILNLSYKQVKTWFQ**
NQRMKSKRWQKNNWPKNSNGVTQAPAPDSM**WRPW**

NANEP7

MWSHPQFEKASDYKDDDDKLAAAVPVK**KQKTRTVFSSTQLCVLNDRFQRQKYLSLQQMQELSNILNLSYKQVKTWFQ**
NQRMKSKRWQKNNWPKNSNGVTQKASAPTYPSLYSSYHQGCLVNPTGNLPMWSNQTWNNSTWSNQTQNIQSWSNHSW
NTQTWCTQSWNNQAWNSPFPAPDSM**WRPW**

Figure S2 (ii). Kuciak et al.

NANEP8

MWSHPQFEKASDYKDDDDKLAAAVPVK**KQKTRTVFSSTQLCVLNDRFQRQKYLSLQQMQELSNILNLSYKQVKTWFQ**
NQRMKSKRWQKNNWPKNSNGVTQGVAPGASAS**MFSIDNILA**ARPRCKDSVLPVAHSAAAPVVFP

NANEP9

MWSHPQFEKASDYKDDDDKLAAAVPVK**KQKTRTVFSSTQLCVLNDRFQRQKYLSLQQMQELSNILNLSYKQVKTWFQ**
NQRMKSKRWQKNNWPKNSNGVTQKASAPTYPSLYSSYHQGCLVNPTGNLPMWSNQTWNNSTWSNQTQNIQSWSNHSW
NTQTWCTQSWNNQAWNSPFAS**MFSIDNILA**ARPRCKDSVLPVAHSAAAPVVFP

NANEP10

MWSHPQFEKASDYKDDDDKLAAAVPVK**KQKTRTVFSSTQLCVLNDRFQRQKYLSLQQMQELSNILNLSYKQVKTWFQ**
NQRMKSKRWQKNNWPKNSNGVTQGVAPGASAS**MFSIDNILA**

NANEP11

MWSHPQFEKASDYKDDDDKLAAAVPVK**KQKTRTVFSSTQLCVLNDRFQRQKYLSLQQMQELSNILNLSYKQVKTWFQ**
NQRMKSKRWQKNNWPKNSNGVTQKASAPTYPSLYSSYHQGCLVNPTGNLPMWSNQTWNNSTWSNQTQNIQSWSNHSW
NTQTWCTQSWNNQAWNSPFAS**MFSIDNILA**

Figure S2 (iii). Kuciak et al.

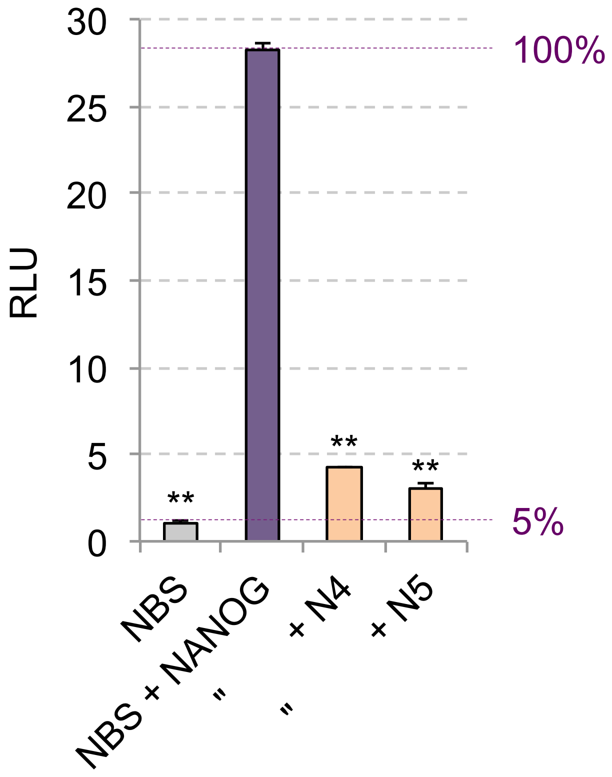

Figure S3. Kuciak et al.

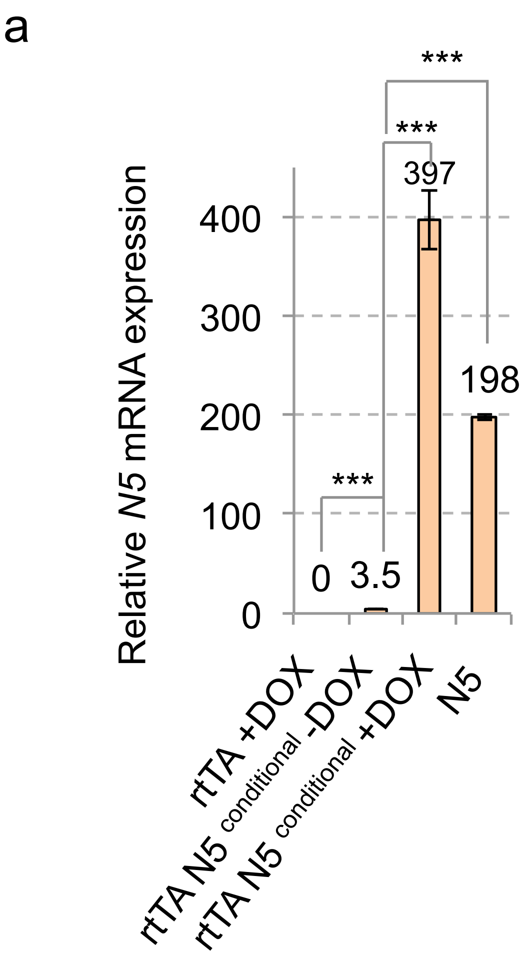

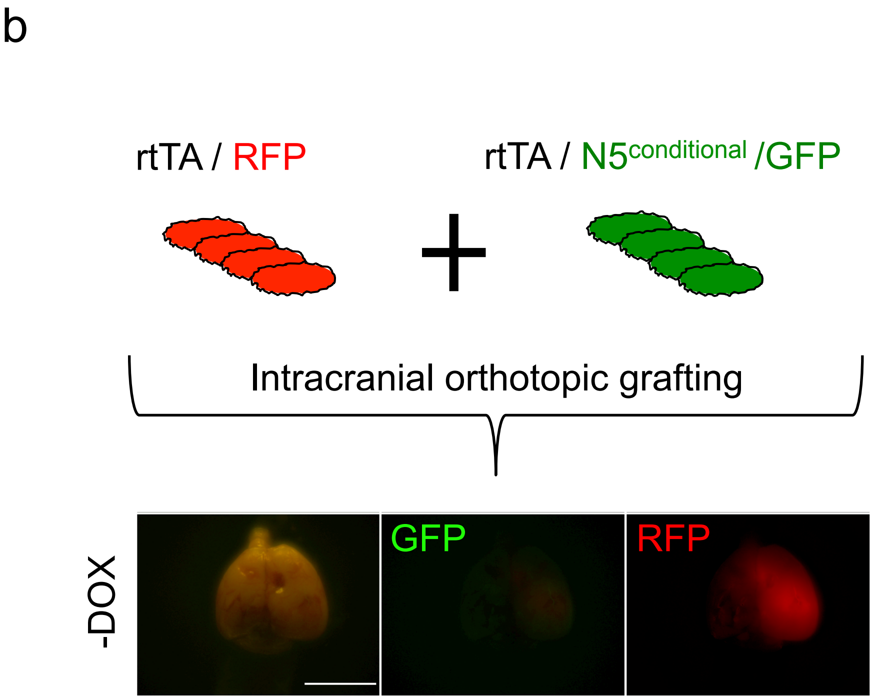

Figure S4. Kuciak et al.

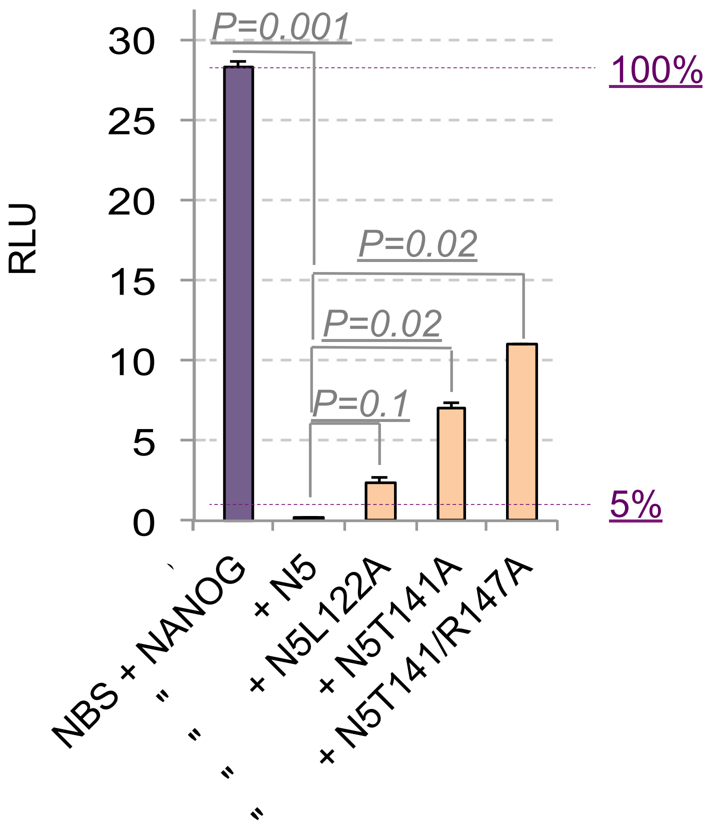

Figure S5. Kuciak et al.

a

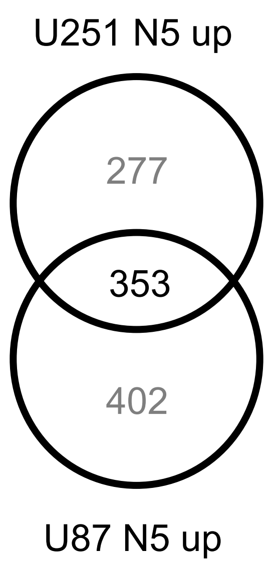

b

Common hits	FC
PTGES	30
TNFAIP6	17
ELN	17
LSP1	14
NGFR	14
GOLGA7B	11
PTGS1	9
CYP26B1	9
ANGPTL4	9
JUP	9
ABI3BP	8
WISP2	8

c

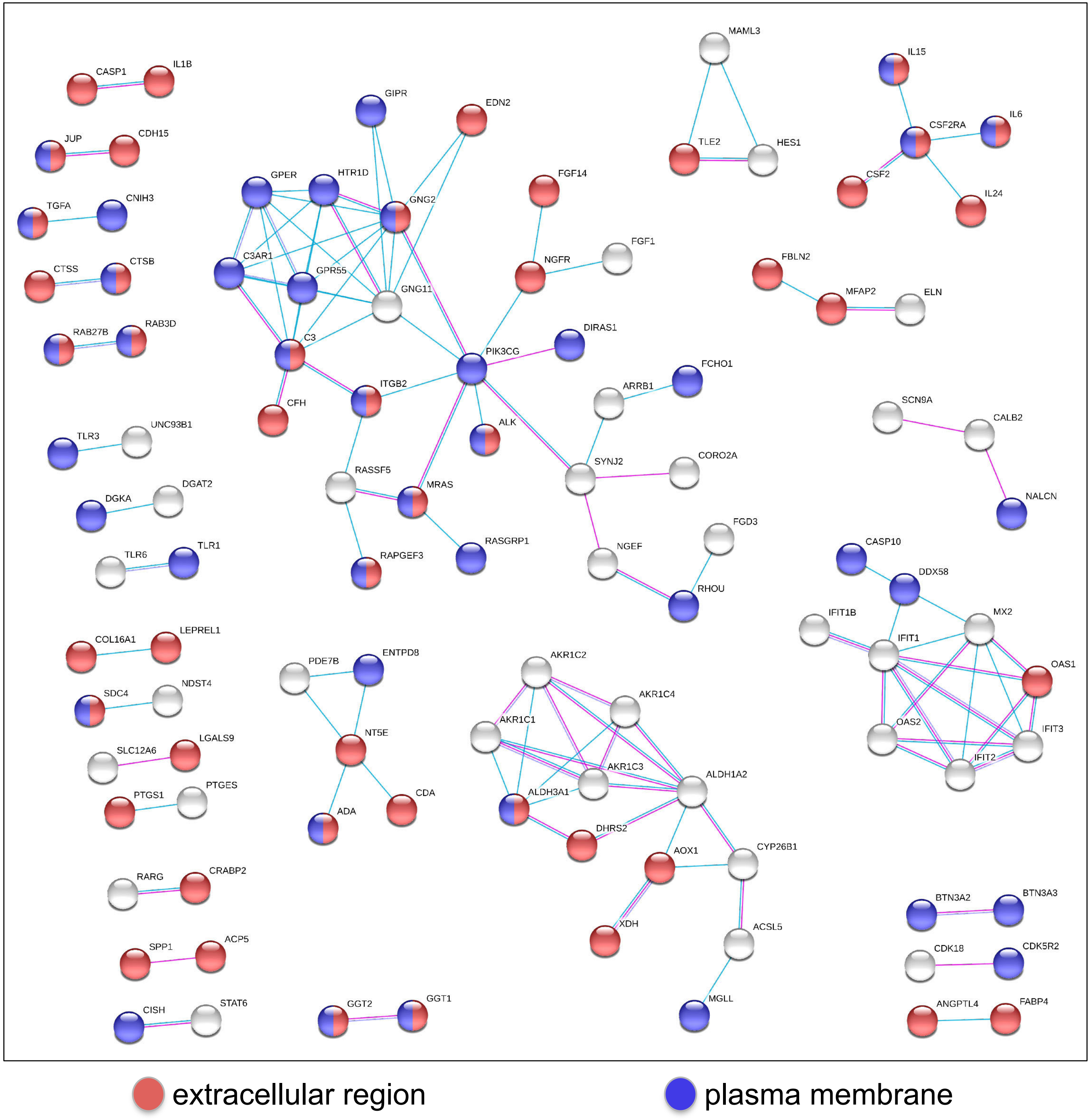

Figure S6 (a-c). Kuciak et al.

d

Ligand Peturbations from GEO up	Adjusted p-value
Interleukin1,beta_human_AMC _GDS4595_ligand:24	1.33E-16
Interleukin_28B_human_hepatocyte _GDS4390_ligand:189	7.39E-13
Interferon_human_keratinocyte _GDS4601_ligand:177	8.66E-13

GO Biological Process 2018	Adjusted p-value
leukotrine D4 biosynthetic process	1.00E-05
leukotrine D4 metbolic process	1.00E-05
doxourbicin metabolic process	1.00E-03
daunorubicin metabolic process	1.00E-03
prostanoid metabolic process	5.00E-04

GO Molecular Function	Adjusted p-value
ketosteroid monooxygenase activity	0.001
bile acid binding	0.001
aldo keto reductase (NADP) activity	0.002

Figure S6 (d). Kuciak et al.

	false discovery rate
**GO Biological Process**	
neurogenesis	1.12E-09
regulation of cell development	1.35E-09
regulation of multicellular organismal process	5.39E-09
cell differentiation	4.67E-09
organ development	1.35E-08
**GO Molecular Function**	
protein binding	8.23E-06
transmembrane receptor protein tyrosine kinase activity	0.0004
receptor binding	0.0039
extracellular matrix structural constituent	0.0039
growth factor binding	0.0252
**GO Cellular Component**	
proteinaceous extracellular matrix	4.97E-05
extracellular matrix	4.97E-05
synapse	5.13E-05
cell surface	6.08E-05
cell junction	0.0001

Figure S7. Kuciak et al.

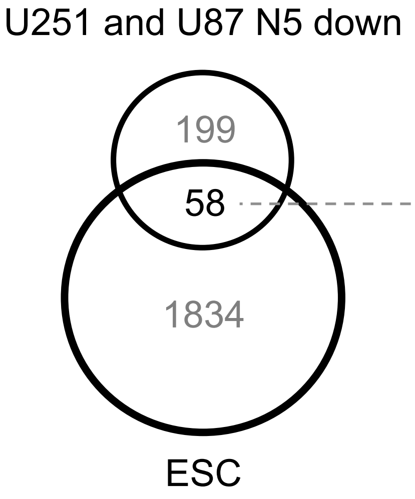

BDNF	*CYP24A1*	*IL17D*	*NTRK2*	*PTGER2*	*TACR1*
BVES	*DACT1*	*IL17RD*	*PAX7*	*RBM24*	*THSD1*
C6orf141	*DLL1*	*IRX1*	*PDE1C*	*RGMA*	*TNFRSF19*
CACNG4	*EPHA5*	*IRX3*	*PDGFRA*	*RGS20*	*TNIK*
CELSR1	*FGF13*	*IRX4*	*PLCB1*	*SFRP1*	*TRIM2*
COL12A1	*GATA3*	*IRX5*	*PLCE1*	*SFRP4*	*TRIM9*
COL14A1	*GDF6*	*KCNH1*	*PPP1R14C*	*SLCO5A1*	*UNC5C*
COL2A1	*GRIN2A*	*MEST*	*PRDM16*	*SOX21*	*WNT3*
CXCL14	*HS3ST3B1*	*NPAS2*	*PRICKLE1*	*STXBP6*	
CXCR4	*HS6ST2*	*NTN1*	*PRRX1*	*SULF2*	

Figure S8. Kuciak et al.

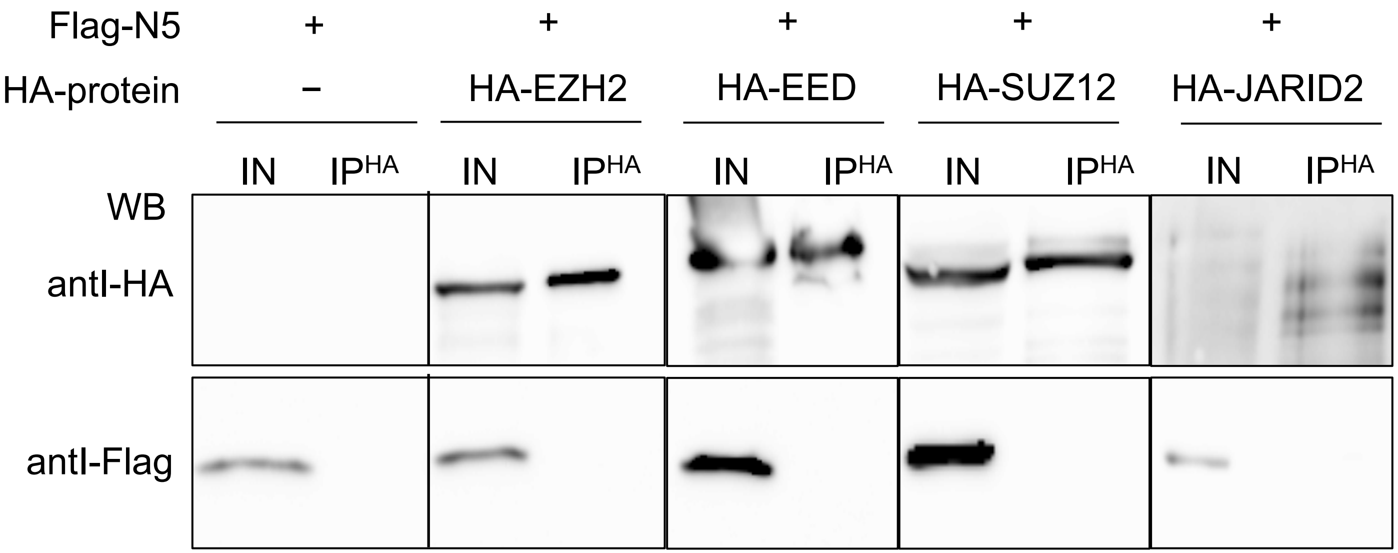

Figure S9. Kuciak et al.
